# Supplementary material for: Assessing the role of regional educational integration policies in fostering university-industry-research innovation: Evidence from China
Source: PLoS One. 2025 Jul 18;20(7):e0325717. doi: 10.1371/journal.pone.0325717 (PMC12273934; doi:10.1371/journal.pone.0325717)
Supplement: Attachment — Submitted filename: comment 11-1.docx [file pone.0325717.s003.docx]

***I am sincerely grateful to the journal’s editor for the opportunity to review the article titled*** *Can regional education integration policy promoted university- industry-research collaborative innovation? –Evidence from China****." The authors have undoubtedly done an excellent job. I have a minor suggestion that could further enhance the quality of the research paper.*** ***Additionally, please provide the revised manuscript with track changes enabled in MS Word.***

**Title Analysis and Coherence Comment**

1. **Title Analysis**: The title reflects an investigation into the role of regional educational integration policies in promoting collaborative innovation between universities, industries, and research institutions. However, it can benefit from a slightly refined structure to highlight the relationship between policy integration and innovation outcomes more clearly. Consider rephrasing to, “Assessing the Role of Regional Educational Integration Policies in Fostering University-Industry-Research Innovation: Evidence from China.”
2. **Coherence Comment**: In assessing the manuscript, ensure the logical flow between sections is clear, linking regional education policies directly with mechanisms that support collaboration across universities, industries, and research entities. For instance, demonstrate how specific policies lead to actionable frameworks or platforms for innovation.

**Introduction**

1. **Introduce Latest Literature**: Begin with a strong theoretical framework around regional education integration and collaborative innovation, connecting it with recent literature.
2. **Enhance Background Context**: Emphasize the significance of collaborative innovation in the Chinese economy and regional policy environment, detailing how education integration is aligned with national innovation goals. Highlight the gap your study addresses in bridging these sectors through structured policies.

**Literature Review**

1. **Summarize Recent Findings**: Using the cited studies, build a foundation that captures the scope of recent collaborative models and the role of educational policy in China.
   - **Key Themes**: Mention themes like the role of educational policy in supporting innovation ecosystems, the influence of economic and technological policies, and cross-sectoral partnerships in fostering sustainable innovation.
2. **Identify Gaps**: Make a clear case for how your paper fills a gap in the existing literature by focusing on China’s unique regional education policy dynamics and their impact on university-industry-research (UIR) collaborations.

**Methodology**

1. **Incorporate Unit Root Tests and Time Effects**: For methodological enhancement, integrate unit root tests to assess the stationarity of variables, which is essential for panel data involving time series to ensure robust estimation.
   - **Suggested Approach**: Use LLC, IPS, ADF-Fisher, or PP-Fisher tests to confirm stationarity of data, especially if dealing with longitudinal data on policy impacts and innovation metrics.
2. **Time Effects Consideration**: Recognize time-fixed effects to account for temporal factors that might influence UIR collaborations (e.g., policy shifts, economic events). This will strengthen the robustness of your results by capturing latent trends over time.

**Suggested Sources**: [*https://doi.org/10.1007/s10668-023-04391-7*](https://doi.org/10.1007/s10668-023-04391-7), <https://doi.org/10.1016/j.heliyon.2024.e33519>,<https://doi.org/10.1556/032.2024.00017>, <https://doi.org/10.1016/j.egyr.2024.08.052>

**Results and Analysis**

1. **Presentation of Findings**: Discuss the impact of regional educational policies on UIR collaborative innovation, drawing clear connections between policy variables and innovation outcomes.
2. **Time Series Insights**: Present unit root test results to establish the consistency of the data, and interpret the time-fixed effects, explaining how they reflect the evolution of policy impacts over time.

**Policy Implications**

1. **Relevant Policy Insights**:
   - **Innovation Ecosystem Support**: Recommend policies to create supportive environments for UIR collaborations, such as funding incentives and platforms for industry-academia partnerships.
   - **Educational Integration Policies**: Encourage enhanced integration of educational institutions into innovation-driven economic frameworks, especially in tech hubs and high-growth regions in China.
   - **Future Directions**: Suggest policies that could further regional integration by focusing on digital transformation in educational frameworks, particularly in fields relevant to the emerging needs of the Chinese economy, such as AI and renewable energy.

**Conclusion and Future Scope**

1. **Summarize Contributions and Limitations**: Briefly summarize how the study contributes to understanding the effects of regional educational policies on collaborative innovation, while noting any limitations in scope or methodology.
2. **Future Scope**: Propose avenues for further research, such as expanding to other Asian economies or assessing the long-term impact of digital educational policies on UIR collaborations.

**Proofreading and Language Precision**

1. **Grammar and Typing Checks**: Ensure that the document is thoroughly proofread for typing errors, grammatical issues, and readability. Utilize tools like Grammarly or Hemingway to catch minor errors and ensure that the language is concise and academic.

By following these structured enhancements, the review report will have a well-defined narrative, greater methodological rigor, and comprehensive coverage of policy implications, making it relevant and impactful for academic and policy audiences alike.
