# Supplementary material for: Assessing the role of regional educational integration policies in fostering university-industry-research innovation: Evidence from China
Source: PLoS One. 2025 Jul 18;20(7):e0325717. doi: 10.1371/journal.pone.0325717 (PMC12273934; doi:10.1371/journal.pone.0325717)
Supplement: Attachment — Submitted filename: Response to Reviewers.doc [file pone.0325717.s004.doc]

Ms. Ref. No.:  PONE-D-24-44621
Title: “*Can regional education integration policy promoted university- industry-research collaborative innovation? –Evidence from China*”
**PLOS ONE**

**Letter by way of reply to Editor and Reviewers**

Dear Editor and Reviewers:

- Thank you very much for your letter and the reviewer's comments concerning our manuscript entitled “Can regional education integration policy promoted university- industry-research collaborative innovation? –Evidence from China”.
- The comments are all valuable and helpful for revising and improving our paper, as well as highlighting the importance of the implications of our studies.
- We have revised the manuscript and would like to re-submit it for your consideration.
- We have addressed the comments raised by the reviewers, and the changes are indicated in the revised manuscript.
- Point-by-point responses to the reviewer’ s comments are listed below this letter.

*****

**The revised manuscript has been thoroughly edited for correct typos, grammar, and syntax.**

***

**We hope that the changes that occurred in the revised manuscript will satisfy the Editor and Reviewers.**

We look forward to your response.

With best wishes！

| ***Referee 1’s comments*** | ***Responses*** |
| --- | --- |
| ***1. Though the findings of the paper is intuitive and reasonable, the methodologies and evidence are questionable, which may not be able to sufficiently support its conclusion. The authors use two-way-fixed-effect difference-in-difference for causal inference. The validity of difference in difference relies on two important assumptions.***  ***The first one is there is no spillover effect, i.e. the control group cannot be impacted by the treatment. However, since treated region is one of the largest economic hubs China. It is unbelievable that the policy will only influence universities and companies in the region but not those in other provinces. The authors also mentioned that there are some collaborations including multiple universities from different provinces, which can be an indication of the potential of spillover effect of treatment.*** | *Thank you very much for your meticulous review and invaluable feedback on our paper. We fully appreciate the importance of methodological rigor and evidential support in academic research, and have therefore taken your concerns to heart.* In response to your concern regarding the potential spillover effect in the difference-in-difference (DID) method, we acknowledge the validity of your point and recognize that it could indeed impact our conclusions. To address this issue more accurately and assess the impact of regional education integration policies on collaborative innovation among universities, industries, and research institutions (UIR) in the Yangtze River Delta (YRD), we have adopted the Regression Discontinuity Design (RDD) approach as per your suggestion. ***Please refer to Pages 19-20 in the revised manuscript.*** The RDD method effectively avoids the interference between treatment and control groups by utilizing a specific threshold (i.e., the "discontinuity") to split the sample, thereby ensuring the accuracy of causal inference. In our study, we conducted RDD separately for the treatment and control groups to ensure that the latter was not "contaminated" by the spillover effects of the policy in the former. The results of the RDD analysis indicate a significant positive policy effect at the discontinuity point for the treatment group, further confirming the effectiveness of the regional education integration policy in promoting UIR innovation cooperation. Meanwhile, the insignificant policy effect at the discontinuity point for the control group suggests that it was indeed unaffected by the policy, thereby ruling out any potential "contamination" from the treatment group. **Table 8 Regression Discontinuity Design Analysis** \| The treatment group \| The control group; VARIABLES \| patent \| patent; lwald \| 0.949*** \| -0.030; \| (5.57) \| (-0.78); Observations \| 64 \| 402 *We are deeply grateful for your constructive feedback, which has prompted us to delve deeper into our methodology and enhance the robustness of our findings.* |
| ***2. The second assumption is the parallel trend assumption. It requires the trend of the treatment and control groups to be parallel over time if there was the treatment. The authors test for the parallel of the pre-treatment trends. Passing the test can only prove half of the parallel trend assumption is correct. Authors should discuss how likely the post-treatment trends would still be parallel without the treatment. Given how important and special YRD region is for China, considering its cumulative advantage in science, technology, education, and governments’ discretion in economic management, it is hard to believe the trend of the region would be the same as the control group without treatment.***  ***What’s more, the claim that the pre-treatment trends being parallel is also not convincing. First, Figure 1 clearly shows the point estimate of the difference between treatment and control is trending up before the treatment. The trend even look consistent to the trend after the treatment. The plot even makes me feel that the after treatment difference is more likely due to the preexisting trend rather than the treatment. Second, even though none of the point estimate in the pre-treatment test is significant at 95% level, the author fails to offer the joint F statistic of the 7 point estimates. When testing for parallel trend, joint F-statistics is more reliable than checking each point estimate. The authors may have realized the flaw in the test. They use PSM to create kernel density weighted average of control groups. However, they failed to show the test for pre-treatment parallel trend for the PSM-DID model.*** | *Thank you very much for your meticulous review and valuable feedback on our paper. We have carefully considered the concerns you raised regarding the parallel trend assumption.*  We fully recognize the importance of the parallel trend assumption in Difference-in-Differences (DID) designs, which requires that the trends of the treatment and control groups would have been parallel in the absence of the treatment. Your point that preliminary tests of pre-treatment trends alone do not fully prove the parallel trend assumption is well taken, and we appreciate your emphasis on this crucial aspect.  To address your concerns more rigorously, we conducted a joint F-test on the pre-treatment time dummy variables, as you suggested. The results of this test, with **an F-statistic of 0.24 and a p-value of 0.9632**, are not significant. This indicates that there were no statistically significant differences in trends between the treatment and control groups before the implementation of the policy, thus supporting the parallel trend assumption. ***Please refer to Pages 15 in the revised manuscript.***  Furthermore, regarding the pre-treatment trend graph (Figure 2) you mentioned, which shows an increasing difference between the treatment and control groups, we have re-examined it. While the graph does show a certain upward trend, the results of the joint F-test suggest that this difference is not statistically significant and therefore does not undermine the parallel trend assumption. Additionally, our RDD (Regression Discontinuity Design) analysis indicates that the policy itself had a significant positive effect, further reinforcing our confidence in the research findings.  Lastly, concerning the pre-treatment parallel trend test for the PSM-DID model, we acknowledge that we did not explicitly present this in the original paper. In the revised version, we have included this analysis and conducted the same joint F-test on the PSM-matched control group, which also supports the parallel trend assumption.  *Thank you once again for your review and valuable comments. We sincerely hope that these revisions adequately address your concerns and enhance the quality of our paper.* |
| ***3. They also attempt to use instrumental variable. But the instruments are problematic, too. First order lag has been widely criticized in academia since it probably violates exclusion restrictions. i.e. instrumental variables can only influence the dependent variable through the corresponding endogenous variables.*** | *Thank you very much for your meticulous review and invaluable feedback on our paper. We have taken your concerns regarding the potential issues with instrumental variables to heart and have made corresponding adjustments to enhance the robustness of our findings.* To bolster the reliability of our results, address potential endogeneity issues, and circumvent the challenges associated with instrumental variable selection in the Two-Stage Least Squares (2SLS) method, we have opted to employ the more efficient and robust System GMM (SYS-GMM) and Difference GMM (DIF-GMM) estimation techniques. This methodological shift aims to accurately capture the relationships among variables while overcoming the limitations of traditional approaches. ***Please refer to Pages 24-25 in the revised manuscript.*** According to the AR serial correlation test results, our model's disturbance terms do not exhibit second-order or higher-order autocorrelation, satisfying the prerequisite assumptions for GMM regression and ensuring the validity of our model. Furthermore, the Hansen over-identification test supports the null hypothesis that "all instrumental variables are valid," further validating the applicability of our model. The regression results reveal a significant positive correlation between the first-order lag of the dependent variable and its current value, highlighting the "time persistence" characteristic of the dependent variable. The GMM method is particularly suited to address such issues. **Table 13 GMM Analysis** \| SYS-GMM \| DIF-GMM; VARIABLES \| patent \| patent; L.patent \| 0.9496*** \| 0.8905***; \| (166.29) \| (82.63); did \| 0.0466*** \| 0.0197**; \| (6.17) \| (2.45); gdp \| 0.0522*** \| 0.0773***; \| (12.24) \| (5.77); urban \| 0.0003 \| 0.0058***; \| (0.61) \| (7.15); gov \| -0.0203 \| -0.2324***; \| (-0.54) \| (-3.72); structure \| 0.0026*** \| 0.0055***; \| (5.72) \| (5.12); gap \| 0.0230 \| 0.4245***; \| (0.84) \| (6.30); fin \| 0.0024 \| -0.0079; \| (0.45) \| (-1.27); Constant \| -0.6055*** \| -1.5444***; \| (-7.64) \| (-6.63); Observations \| 431 \| 431; Number of id \| 31 \| 31; AR1 \| 0.002 \| 0.001; AR2 \| 0.828 \| 0.785; Hansen \| 0.524 \| 0.681 *We sincerely appreciate your guidance and acknowledge that these modifications have not only improved the quality of our paper but also deepened our understanding of the research question.* |
| ***4. I also observed some mistakes in the description of concepts and methods. For instance, between Row 282 and 285, “if DIDk−7 to DID−2 is significantly different from it zero, then the model is able to satisfy the parallel trend assumption”. This is clearly opposite to the truth.*** | *Thank you very much for your meticulous review of our paper and for providing invaluable feedback. We particularly appreciate your pointing out the error in the description of concepts and methods, specifically regarding the statement about the parallel trend assumption between lines 282 and 285. We deeply regret any confusion this may have caused and sincerely apologize for the mistake.*  We have now revised this error in Row 298 in the revised manuscript. Upon careful examination, we acknowledge that the original wording indeed conveys the opposite of what is intended. The correct interpretation of the parallel trend assumption should be that if the DID estimates (from DIDk−7 to DIDk−2) are not significantly different from zero, it indicates that the trends in the treatment and control groups during these periods are parallel, thus supporting the validity of the model.  *Furthermore, we will conduct a thorough review of the entire paper to ensure that all related statements are accurate and adhere to academic rigor. We will also enhance the explanation of the concepts and methods section to ensure clarity for our readers regarding our research design and analytical approach.*  *Once again, thank you for your valuable guidance. Your feedback is instrumental in improving the quality of our work.* |
| ***Referee 2’s comments*** | ***Responses*** |
| 1. ***In the introduction – the organization of the paper.*** | *Thank you very much for your valuable comments, especially regarding the "organization of the introduction" of the paper. Your feedback has been instrumental in helping us improve the structure and content of our manuscript. In the revised version, we have optimized the organization of the introduction to ensure clarity, comprehensiveness, and alignment with the study’s objectives.*  ***Please refer to Pages 2-3 in the revised manuscript.*** *The following adjustments have been made:*  **1.Research Background**: At the beginning of the introduction, we discuss the global importance of innovation for economic development and sustainable growth, highlighting the critical role of university-industry-research (UIR) collaborative innovation as a key component of regional innovation systems. We also emphasize the importance of regional education integration policies in fostering innovation globally, particularly in emerging economies.  **2.Research Significance and Issues**: We identify key challenges in UIR collaborative innovation, such as fragmented policies, unequal resource distribution, and the lack of effective collaboration platforms. These challenges are particularly prevalent in emerging economies like China, where government-driven regional education policies play a unique role. This section illustrates the necessity of studying how such policies influence innovation outcomes.  **3.Literature Integration and research gaps**: We integrate international experiences (e.g., practices from the EU, OECD, and Nordic countries), emphasizing their relevance to regional education integration and innovation. However, we highlight gaps in the existing research, such as the lack of quantitative analysis, limited focus on dynamic interactions, and insufficient understanding of how these policies perform in different economic and social contexts, particularly in developing countries like China.  **4. Research Region and Objectives**: The introduction then focuses on the Yangtze River Delta (YRD) in China, a region with a strong economic foundation and abundant educational resources, making it an ideal case for studying regional education integration policies. We clearly define our research objectives:  *To analyze the impact of regional education integration policies on UIR collaborative innovation using a Difference-in-Differences (DID) model.  *To investigate the heterogeneous effects of these policies across provinces and levels of innovation intensity.  *To explore the mediating mechanisms of endogenous factors, such as human capital, government support, economic capital, and social stability.  **5. Contributions**: Lastly, we summarize the contributions of the study. Theoretically, it bridges the gap in understanding how education policies influence innovation in emerging economies. Practically, it provides policy recommendations for optimizing regional education integration to promote collaborative innovation.  These adjustments ensure that the introduction provides a logical flow from the research background and significance to the study’s objectives and contributions, effectively addressing your comments.  *Thank you again for your insightful feedback, which has significantly improved the clarity and focus of our manuscript.* |
| 2. ***The literature review is very simplistic, because the hypotheses are focused on specific notions related to capital (economic and intellectual) or social indicators. Thus, the literature review does not cover/support the problem under discussion.*** | *Thank you very much for your valuable feedback regarding the literature review section of our manuscript. We have carefully considered your comments and made significant improvements to address the issues raised.*  ***Please refer to Pages 3-11 in the revised manuscript.*** *Below are the specific actions we have taken:*  **1. Restructuring the Literature Review**  The literature review has been reorganized into five interconnected sections to ensure logical coherence and comprehensive coverage of the research problem:  **Section 2.1 Definition of Regional UIR Collaborative Innovation**: Provides a conceptual definition of UIR innovation and highlights its practical challenges and opportunities, setting the foundation for understanding the research problem.  **Section 2.2 The Impact of Regional Education Policies on Regional Collaborative Innovation**: Summarizes international practices and empirical evidence, providing a comparative perspective on how regional education policies foster innovation globally.  **Section 2.3 Regional Education Integration Policy and UIR Collaborative Innovation in the Yangtze River Delta**: Focuses on China’s unique regional context, particularly the disparities in economic and educational resources across provinces, and their impact on the effectiveness of integration policies.  **Section 2.4 Summary**: Synthesizes key insights from the previous sections, identifies research gaps (e.g., the limited understanding of policy impacts in developing economies like China), and justifies the study’s focus on the Yangtze River Delta. This section bridges the literature review with the hypotheses and research questions.  **Section 2.5 Research Hypotheses**: Articulates hypotheses that connect the literature review with the research focus and Chinese context, ensuring that each hypothesis is grounded in theoretical, empirical, and contextual discussions.  **2. Understanding of UIR innovation ( Section 2.1)**  In **Section 2.1**, we define the concept of regional UIR collaborative innovation and highlight its core purposes, challenges, and practical dimensions. This section provides the foundational understanding of the research topic, focusing on the practical implications and key barriers (e.g., resource allocation and cultural differences) that hinder collaborative innovation. It sets the stage for subsequent sections, where theoretical frameworks and empirical evidence are discussed in greater depth (Sections 2.2 and 2.3).  **3. International Practices and Evidence (Section 2.2)**  In **2.2 The Impact of Regional Education Policies on Regional Collaborative Innovation**, we comprehensively analyzed global practices and empirical evidence to provide a comparative perspective on how regional education policies foster UIR collaboration. Key discussions include:  **Theoretical Foundations**: Building on theories such as Growth Pole Theory, Synergetics Theory, and Innovation Theory, we emphasize the critical role of regional policies in coordinating innovation entities and promoting collaborative innovation.  **Global Practices and Case Studies**: **Germany**: Case studies demonstrate how research universities and application-oriented universities collaborate with local industries to enhance innovation capacity. **UK Cluster Policy Steering Group**: Highlights the role of industrial clusters and knowledge transfer in promoting commercialization of research achievements. **Kyoto Innovation Belt (Japan)**: Illustrates how government guidance aligns university research with industrial needs to drive innovation. **REGLAB Model (Nordic Countries)**: Showcases how regional innovation ecosystems are supported through collaborative platforms and services.  **Challenges and Heterogeneity**: Discussing the diverse impacts of regional policies, we highlight challenges such as resource monopolization (e.g., institutional hijacking) and uneven innovation efficiency due to variations in regional economic strength, industrial structure, and academic disciplines.  **Policy Mechanisms**: Exploring mechanisms like financial incentives, platform construction, and intellectual property protection, we provide insights into how governments facilitate UIR collaboration and address implementation challenges.  These findings provide a comparative framework for evaluating and improving China’s regional education policies, offering valuable lessons for enhancing UIR collaborative innovation.  **4. China’s Context and Challenges (Section 2.3)**  In 2.3 Regional Education Integration Policy and UIR Collaborative Innovation in the Yangtze River Delta, we provided a comprehensive analysis of China’s unique socio-economic and policy environment. This section emphasizes:  Regional Advantages and Progress: Highlighting the YRD's strong economic and educational foundations, the success of policies like the 2014 Guidelines, and examples of UIR collaborative innovation projects, such as industrial parks in Nanjing and Hangzhou.  Policy Challenges: Addressing the existing gaps in regional innovation cooperation, such as resource allocation disparities and fragmented policy implementation.  Implications for Research: These discussions form the basis for investigating the heterogeneity in policy effects and justify the study’s focus on the Yangtze River Delta.  **5. Hypotheses Development Based on the Literature (Section 2.5)**  In **2.5 Research Hypotheses**, we systematically connected the literature review to the hypotheses to ensure logical consistency and establish a strong theoretical and contextual foundation.  The hypotheses are grounded in established theories and supported by both international evidence and the specific context of China. For example, **H2a** is based on Regional Disparities Theory, which highlights how differences in economic and educational resources shape policy outcomes, as demonstrated by the contrasts between resource-rich cities like Jiangsu and resource-scarce areas such as Anhui. Similarly, **H2b**, supported by Innovation Spillover Theory, examines how regional policies differentially impact disruptive and incremental innovations. Furthermore, the mediating factors in **H3a–H3d**, including human capital, government support, economic capital, and social stability, are firmly rooted in theoretical frameworks such as Human Capital Theory and Institutional Theory, ensuring a robust conceptual foundation.  To align these hypotheses with the literature review, **Section 2.3** provides an in-depth discussion of resource disparities in the Yangtze River Delta, which directly supports **H2a** and **H2b**. Meanwhile, **Section 2.2** draws on international practices, highlighting mechanisms like government funding and collaborative platforms, which underpin the mediating factors explored in **H3a–H3d**.  Finally, the hypotheses are contextualized within China’s unique socio-economic and policy environment. The government-driven approach to education integration and the regional disparities in the Yangtze River Delta provide a practical basis for the hypotheses, demonstrating their relevance to the research problem and the broader Chinese context. |
| 3. ***First, the hypotheses should be proposed after the sub-chapter 2.4 because they seem not related to China and I understand that the sample is based on a very specific region in the country*** | *Thank you for your valuable feedback. We have revised the manuscript to address your concern regarding the placement and contextual relevance of the hypotheses.* ***Please refer to Pages 8-11 in the revised manuscript.***  **1.Hypotheses Positioned After Sub-Chapter 2.4**  The hypotheses section has been placed after sub-chapter 2.4 Summary, which synthesizes insights from the literature and identifies research gaps related to China’s regional education policies and UIR collaborative innovation. This ensures a logical transition from the literature review to the hypotheses.  **2.Linking Hypotheses to the Chinese Context**  The hypotheses are specifically grounded in the Yangtze River Delta (YRD) region, a representative area in China. For example:  H1 highlights the role of government-led education policies in promoting UIR collaboration, as exemplified by the YRD’s resource integration and joint innovation programs.  H2a and H2b reflect the significant regional disparities in economic and educational resources within the YRD, such as differences between Jiangsu and Anhui.  H3a–H3d focus on mediating factors like human capital and government support, contextualized within China’s socio-economic environment.  These revisions clarify that the hypotheses are directly related to China and tailored to the specific characteristics of the YRD region. |
| 4. ***The discussion part is missing, in my opinion. The study is based on a specific region in China, but why is it relevant for other countries or regions?*** | *Thank you for your valuable feedback regarding the discussion section. We have carefully addressed your comments and made significant revisions to include a comprehensive discussion, which was previously missing from the manuscript. The newly added discussion section not only contextualizes the findings within the Yangtze River Delta (YRD) region in China but also explores their broader implications for other countries and regions.*  ***Please refer to Pages 26-28 in the revised manuscript.*** *Below are the key improvements made:*  **1. Addressing the Missing Discussion Section**  The discussion section has been newly added to ensure that the findings are fully interpreted and their implications are thoroughly analyzed. This addition directly addresses your concern about the lack of discussion in the original manuscript.  The discussion now provides a detailed interpretation of the empirical results, linking them to relevant theories and global cases. This ensures that the findings are grounded in a robust theoretical framework, demonstrating their significance beyond the regional context.  **2. Contextualizing Findings Within the Yangtze River Delta**  The discussion highlights how regional education integration policies in the YRD enhance collaboration among universities, industries, and research institutions. This collaboration has been shown to strengthen regional innovation systems, improve innovation capacity, and optimize resource allocation.  Specific findings, such as the heterogeneity of economic levels and innovation intensity, are contextualized within the unique socio-economic conditions of the YRD, providing a deeper understanding of policy impacts in this region.  **3. Relevance to Other Countries and Regions**  The discussion explicitly connects the findings to global contexts, demonstrating their applicability beyond the YRD. For instance, international studies (e.g., OECD, 2017; Lindqvist, 2013) have similarly shown that policies promoting university-industry collaboration drive regional innovation in countries such as Germany, Japan, and Ireland.  Parallels are drawn between the YRD and globally recognized innovation hubs like Silicon Valley, illustrating how education integration policies can drive technological innovation and economic growth across diverse economic and cultural settings.  **4. Broader Policy Implications**  The discussion also identifies universal drivers of innovation—human capital, government support, economic capital, and social stability—and situates these within a multidimensional framework. These factors are essential components for fostering innovation and have been widely recognized in global research (e.g., Isaksen, 2017; Fitjar, 2014).  By examining these drivers in the context of the YRD, the study provides valuable insights for policymakers worldwide, offering practical guidance for designing and implementing effective innovation policies in various regional settings. |
| 5. ***The results part does not refer to hypotheses being validated or not.*** | *Thank you for your insightful comment. In the revised manuscript, we have ensured that each hypothesis is explicitly referenced in the discussion section.* ***Please refer to Pages 26 in the revised manuscript.***  The first, second, third, and fourth paragraphs of the discussion section directly state whether Hypotheses 1, 2a, 2b, and 3 are supported or not. Each paragraph begins with a clear statement about the validation of the respective hypothesis and is accompanied by corresponding empirical evidence from Tables 4, 10, 11, and 12.  This structure ensures that the relationship between the results and the hypotheses is explicitly addressed, providing clarity and consistency. |
| 6. ***The paper needs a better substantiation on existing literature and discussion which makes it relevant for other authors, given that the sample is based on a specific region in China*** | *Thank you for your insightful comment. We have carefully addressed your concerns by revising the manuscript to strengthen its substantiation on existing literature and expand the discussion, ensuring its relevance for other authors and broader academic contexts. Below are the specific actions we have taken:*  **1. Expanding the Literature Review**  In the revised manuscript, we have expanded the literature review to include a more comprehensive discussion of international studies on regional education policies and their role in fostering innovation. For example:  Studies from the OECD (2017), Lindqvist (2013), and Daimer et al. (2017) have been incorporated to provide a comparative perspective on how education policies promote university-industry collaboration globally.  We have highlighted how our study extends the existing literature by focusing on emerging economies, specifically the Yangtze River Delta (YRD) in China, where the socio-economic context and policy environment differ significantly from those in developed countries.  These additions ensure that the literature review connects our findings to global discussions while highlighting the unique contributions of this study.  **2. Enhancing the Discussion Section**  The discussion section has been revised to emphasize the broader applicability of the findings. Specifically in two sections:  The mechanisms of regional education integration policies—such as fostering collaboration between universities and industries, addressing regional disparities, and promoting innovation outputs—are discussed in the context of their relevance to other countries and regions.  International examples, such as Germany, and Japan, are referenced to demonstrate the global applicability of the findings. For instance, parallels between the YRD and these innovation hubs are drawn to highlight how similar policies can drive innovation in different economic and cultural settings.  These revisions clarify how the findings provide insights that are not limited to the YRD but also applicable to broader regional innovation systems.  **3. Clarifying Contributions in the Introduction and Conclusion**  To further align the manuscript with your suggestions, we have revised the introduction and conclusion to explicitly state the study’s contributions to global literature and its relevance to other contexts.  In the Introduction: We have emphasized the international significance of the research problem and how this study addresses key gaps in the understanding of regional education integration policies in emerging economies.  In the Conclusion: We have summarized the study’s broader implications, highlighting how the findings on policy mechanisms, regional disparities, and key drivers (e.g., human capital, government support) offer practical guidance for optimizing innovation policies in other regional contexts worldwide. |
| ***Referee 3’s comments*** | ***Responses*** |
| ***Title Analysis and Coherence Comment***  ***1. Title Analysis: The title reflects an investigation into the role of regional educational integration policies in promoting collaborative innovation between universities, industries, and research institutions. However, it can benefit from a slightly refined structure to highlight the relationship between policy integration and innovation outcomes more clearly. Consider rephrasing to, “Assessing the Role of Regional Educational Integration Policies in Fostering University-Industry-Research Innovation: Evidence from China.*** | *Thank you for your insightful suggestion regarding the title of the manuscript. We have carefully considered your feedback. We agree that the revised title better highlights the relationship between policy integration and innovation. Accordingly, we have updated the title to:*  **“Assessing the Role of Regional Educational Integration Policies in Fostering University-Industry-Research Innovation: Evidence from China.”**  *This revised title provides a clearer focus on the study's key themes and aligns with the purpose and findings of the research. We sincerely appreciate your constructive input, which has helped us improve the clarity and precision of the manuscript.* |
| ***Title Analysis and Coherence Comment***  ***2. Coherence Comment: In assessing the manuscript, ensure the logical flow between sections is clear, linking regional education policies directly with mechanisms that support collaboration across universities, industries, and research entities. For instance, demonstrate how specific policies lead to actionable frameworks or platforms for innovation.*** | *Thank you for your insightful comment regarding the logical flow and connection between regional education policies and actionable mechanisms for innovation. We have carefully addressed your suggestions by making the following revisions to the manuscript:*  **1.Enhancing Logical Flow Across Sections**  The introduction and literature review have been revised to clearly establish the link between regional education policies and specific mechanisms that support collaboration. These mechanisms are presented as actionable frameworks through which policies influence innovation.  Throughout the results and discussion sections, we ensure a consistent narrative that connects empirical findings to these mechanisms, providing a logical flow between policy objectives, implementation, and measurable outcomes.  **2.Section-Level Revisions**  Introduction and Literature Review: Now explicitly outline how regional education policies foster actionable frameworks, supported by international and domestic studies.  Results: Clearly link empirical findings to specific mechanisms driven by policys in Table 12.  Discussion: Further analyze the implications of these mechanisms, demonstrating their relevance to global innovation systems and providing actionable insights for policymakers.  *These revisions strengthen the manuscript’s coherence and highlight the practical pathways through which policies foster innovation collaboration. Thank you again for your valuable feedback, which has greatly improved the clarity and logical consistency of the manuscript.* |
| ***Introduction***  ***1. Introduce Latest Literature: Begin with a strong theoretical framework around regional education integration and collaborative innovation, connecting it with recent literature.*** | *Thank you for your thoughtful suggestion regarding the introduction. In the revised manuscript, we have carefully incorporated a strong theoretical framework and integrated the latest literature to establish a solid foundation for the study.* ***Please refer to Pages 2-3 in the revised manuscript.*** *Below are the specific improvements made to address your comment:*  **1.Incorporation of a Strong Theoretical Framework**  The revised introduction explicitly situates the study within three key theoretical perspectives: Growth Pole Theory, Synergetics Theory, and Innovation Theory. These theories collectively provide a robust framework for analyzing the role of regional education integration policies in fostering UIR collaborative innovation. For example, Growth Pole Theory emphasizes the role of economic core areas in driving regional innovation, while Synergetics Theory highlights the importance of coordination among innovation actors, and Innovation Theory explains how policies stimulate technological progress and enhance regional innovation capacity. These perspectives offer a theoretical foundation that connects policy mechanisms to innovation outcomes.  **2.Connection to Latest Literature**  We have integrated recent international and domestic studies on regional education policies and UIR collaboration. For example, studies from the OECD (2017) and Witte (2006) demonstrate the role of regional education policies in optimizing resource allocation and facilitating cross-sector collaboration in developed economies. We also highlight gaps in the existing literature, including the lack of focus on emerging economies like China, the limited understanding of dynamic interactions within innovation systems, and the heterogeneity of policy impacts (Rodríguez-Pose, 2015; Knight, 2016). By situating our study within these discussions, we address the global relevance of our research question.  **3.Relevance to China and Emerging Economies**  The revised introduction emphasizes the unique context of China, particularly the Yangtze River Delta (YRD), as a key region for implementing regional education integration policies. We discuss how China’s government-driven approach contrasts with the market-driven policies of Europe and the United States, highlighting the regional disparities in policy implementation and their effects on innovation outcomes. This connection underscores the significance of studying China as an emerging economy to fill the gap in global literature and provides a framework for extending insights to other developing regions.  **4.Clear Articulation of Research Objectives and Contributions**  The introduction concludes by clearly stating the study’s objectives and methodological approach, including the use of a Difference-in-Differences (DID) model to quantitatively assess the impact of regional education integration policies in the YRD. The study also explores heterogeneity in policy effects and examines mediating factors such as human capital, government support, economic capital, and social stability. These contributions are positioned to provide actionable insights for policymakers in China and other emerging economies.  *These revisions ensure that the introduction is grounded in a strong theoretical framework, incorporates the latest literature, and highlights the global and regional significance of the study. Thank you again for your constructive feedback, which has greatly improved the clarity and rigor of the introduction.* |
| ***Introduction***  ***2. Enhance Background Context: Emphasize the significance of collaborative innovation in the Chinese economy and regional policy environment, detailing how education integration is aligned with national innovation goals. Highlight the gap your study addresses in bridging these sectors through structured policies.*** | *Thank you for your insightful comment regarding the introduction. We have carefully revised this section to address your suggestions.*  ***Please refer to Pages 2-3 in the revised manuscript.*** *The updated introduction now emphasizes the following key aspects:*  **1.Significance of Collaborative Innovation:**  The revised introduction highlights the critical role of collaborative innovation (UIR) in enhancing China’s economic competitiveness and driving technological progress. We discuss how regional education integration policies align with national innovation goals, focusing on human capital development, resource optimization, and regional coordination.  **2.Research Gap and Contributions:**  We explicitly identify gaps in existing research, particularly the limited understanding of how education integration policies function in emerging economies like China. The introduction highlights the lack of quantitative studies on UIR collaboration outcomes and heterogeneity analysis, positioning this study as a significant contribution to bridging these gaps.  **3.Contextual and Theoretical Foundations:**  The revised section integrates theoretical frameworks, including Growth Pole Theory, Synergetics Theory, and Innovation Theory, to provide a robust foundation for understanding the role of policies in fostering innovation. These theories offer valuable insights into how regional education policies can address structural barriers to innovation and promote cross-sector collaboration.  **4.Study Design and Relevance:**  The updated introduction outlines the study’s focus on the Yangtze River Delta, a representative region for analyzing the impact of education policies in China. It also describes the application of the Difference-in-Differences (DID) model to assess policy impacts and explore regional disparities, ensuring the research is methodologically rigorous and contextually relevant.  *We believe these revisions address your suggestions by providing a stronger background context, emphasizing the alignment of education integration with national innovation goals, and clearly articulating the research gap and contributions. Thank you again for your valuable feedback, which has significantly improved the clarity and relevance of the introduction.* |
| ***Literature Review***  ***1.Summarize Recent Findings: Using the cited studies, build a foundation that captures the scope of recent collaborative models and the role of educational policy in China.***  ***Key Themes: Mention themes like the role of educational policy in supporting innovation ecosystems, the influence of economic and technological policies, and cross-sectoral partnerships in fostering sustainable innovation.*** | *Thank you for your valuable suggestions regarding the literature review section. In response to your comments, we have comprehensively revised this section to address the identified issues.* ***Please refer to Pages 3-11 in the revised manuscript.*** *Below are the specific improvements made:*  **1.Summarizing Recent Findings:**  We have incorporated recent findings from both global and Chinese contexts to build a solid foundation for understanding collaborative models and the role of educational policies. For example, Section 2.2 highlights international practices (e.g., Germany, the UK, Japan) in fostering UIR collaboration, while Section 2.3 contextualizes these insights within China’s unique regional policy environment, particularly in the Yangtze River Delta.  **2. Addressing Key Themes:**  The revised review emphasizes three key themes:  a. The role of educational policies in supporting innovation ecosystems through talent development, resource sharing, and platform creation.  b. The influence of economic and technological policies on regional collaboration, such as the integration of education and industry to drive innovation clusters.  c. The importance of cross-sectoral partnerships, demonstrated through examples like the establishment of industrial parks and collaborative innovation hubs in Nanjing and Hangzhou.  **3.Highlighting Research Gaps and Contributions:**  The revised summary (Section 2.4) explicitly identifies gaps in the existing literature, such as the lack of quantitative studies on UIR collaboration outcomes, insufficient focus on dynamic interactions, and limited exploration of policy heterogeneity in emerging economies like China. Our study addresses these gaps through a DID model, providing empirical insights into the effects of regional education policies on innovation cooperation, with a focus on patent collaboration.  These revisions ensure that the literature review provides a comprehensive foundation for the study while directly addressing your suggestions.  *Thank you again for your constructive feedback, which has greatly enhanced the clarity and relevance of this section.* |
| ***Literature Review***  ***2. Identify Gaps: Make a clear case for how your paper fills a gap in the existing literature by focusing on China’s unique regional education policy dynamics and their impact on university-industry-research (UIR) collaborations.*** | *Thank you for your valuable feedback and suggestions on our manuscript. We have carefully considered your comments and made the corresponding revisions to the paper. In particular, regarding the literature review and the explanation of how our paper fills gaps in the existing literature, we added 2.4 Summary section to clearly identify gaps.* ***Please refer to Pages7 in the revised manuscript.*** *It includes the followings:*  **Research method:** In the revised manuscript, we explicitly state that although existing research has explored the impact of regional education policies on university-industry-research (UIR) collaborative innovation, most studies have not used quantitative methods to directly measure the tangible outcomes of UIR cooperation, nor have they sufficiently analyzed the dynamic interactions among collaborating regions. To address this gap, we employed the Difference-in-Differences (DID) model to quantitatively assess the impact of regional education integration policies on UIR collaboration, specifically focusing on patent cooperation quantity (i.e., patents co-applied by universities, research institutions, and enterprises across provinces and cities). This approach fills the gap in quantitative analysis in the current literature.  **Mediating Role of Internal Driving Factors:** We also strengthened the discussion on the mediating role of internal driving factors in the implementation of regional policies, noting that there is a lack of systematic analysis in the existing literature. In the revised manuscript, we provide a more detailed explanation of the role of these factors in mediating the relationship between education policies and innovation cooperation.  **Regional Differences and Policy Effectiveness:** We further addressed the challenges faced by regions with different economic foundations, educational resources, and policy execution capacities. While some studies mention regional differences in policy effectiveness, there has been limited in-depth exploration of the specific challenges faced by regions with different socio-economic conditions. We expanded this section in our revision to ensure a more comprehensive discussion of these issues from both theoretical and practical perspectives.  **China's Unique Context:** We also emphasized that, while much of the current literature focuses on policy practices in developed countries, research on China, a developing country, is relatively scarce, especially regarding how to address the challenges of different socio-economic contexts. Our study fills this gap by exploring China's regional education policies and their international applicability.  *We believe these revisions enhance the academic contribution of the paper and more clearly demonstrate how our research addresses gaps in the existing literature. Thank you once again for your thoughtful review and constructive feedback. We look forward to your further comments.* |
| ***Methodology***  ***1. Incorporate Unit Root Tests and Time Effects: For methodological enhancement, integrate unit root tests to assess the stationarity of variables, which is essential for panel data involving time series to ensure robust estimation.***  ***Suggested Approach: Use LLC, IPS, ADF-Fisher, or PP-Fisher tests to confirm stationarity of data, especially if dealing with longitudinal data on policy impacts and innovation metrics.*** | *Thank you very much for your thorough review of our paper and for the valuable suggestions you have provided. We highly appreciate your emphasis on incorporating unit root tests to assess the stationarity of variables, which is indeed crucial for ensuring robust estimations in panel data involving time series. In response to your comments, we have made the necessary revisions to our methodology.* ***Please refer to Page 12-13 in the revised manuscript.*** Specifically, we conducted the ADF (Augmented Dickey-Fuller) unit root test to evaluate the stationarity of each variable in our dataset. The results indicated that some variables were not stationary in their original form. Therefore, we applied first-difference transformation to these variables and re-conducted the ADF test. We are pleased to report that all first-differenced variables rejected the null hypothesis, indicating that they are all stationary at the first difference, thus ensuring the stationarity of our data for subsequent regression analysis. **Table 2 Augmented Dickey-Fuller (ADF) unit root test** Variable \| Level \| ADF statistic \| level \| ADF statistic \| result; patent \| origin \| -1.5235 \| First difference \| 17.3713*** \| stationary; gdp \| origin \| 14.4690*** \| First difference \| 14.2256*** \| stationary; urban \| origin \| -0.3672 \| First difference \| 15.6548*** \| stationary; gov \| origin \| 12.8064*** \| First difference \| 14.3991*** \| stationary; structure \| origin \| 5.4705*** \| First difference \| 11.7034*** \| stationary; Gap \| origin \| 7.2679*** \| First difference \| 16.3939*** \| stationary; Fin \| origin \| 4.4091*** \| First difference \| 26.5543*** \| stationary Furthermore, considering that not all original sequences were stationary, we also performed the EG two-step cointegration test to verify the existence of long-term cointegration relationships among the variables. The test results showed that the residual series was stationary, confirming the presence of cointegration among the variables. **Table 3 Engle-Granger (EG) two-step cointegration test** Variable \| Level \| ADF statistic \| result; e \| origin \| -4.051*** \| stationary *We have detailed these testing methods, procedures, and results in the revised version of our paper, and made necessary adjustments to the relevant sections to enhance the methodology. We sincerely thank you for your guidance and suggestions, which have not only improved the quality of our paper but also greatly benefited our research.* |
| ***Methodology***  ***2. Time Effects Consideration: Recognize time-fixed effects to account for temporal factors that might influence UIR collaborations (e.g., policy shifts, economic events). This will strengthen the robustness of your results by capturing latent trends over time.***  ***Suggested Sources: https://doi.org/10.1007/s10668-023-04391-7, https://doi.org/10.1016/j.heliyon.2024.e33519,https://doi.org/10.1556/032.2024.00017, https://doi.org/10.1016/j.egyr.2024.08.052*** | *Thank you very much for your insightful comments and suggestions on our paper, particularly regarding the consideration of time-fixed effects to account for temporal factors that may influence UIR collaborations. We fully recognize the importance of capturing latent trends over time to strengthen the robustness of our results.* In response to your feedback, we have conducted a thorough analysis of time trend effects. ***Please refer to Page 20-21 in the revised manuscript.*** Specifically, we employed two methods to address this issue. First, we included a time trend term as a control variable in our regression model. The results showed that the DID (Difference-in-Differences) estimator remained significant even after accounting for the time trend, indicating that our findings are robust to the influence of temporal factors. Second, we interacted the DID estimator with multiple year dummy variables and included these interaction terms as control variables in the regression. The results once again confirmed the validity of the DID estimator, further supporting the robustness of our findings. The empirical results, as presented in the table below, clearly demonstrate the significance of the DID estimator in both models, along with other control variables. The adjusted R-squared values also indicate a good fit of the models. **Table 9 Time Trend Effects Analysis** \| With Time Trend Effects \| Trend Decomposition of DID; VARIABLES \| patent \| patent; did \| 0.331*** \| 0.508***; \| (5.50) \| (12.05); gdp \| 0.215*** \| 0.236***; \| (12.72) \| (4.04); urban \| 0.011 \| 0.012; \| (1.64) \| (1.67); gov \| -0.828 \| -0.701; \| (-1.47) \| (-1.29); structure \| 0.009*** \| 0.008***; \| (3.17) \| (3.20); gap \| 2.864*** \| 2.729***; \| (4.56) \| (5.03); fin \| -0.113*** \| -0.085***; \| (-3.86) \| (-3.37); Constant \| -173.091*** \| -4.443***; \| (-8.82) \| (-4.02); Observations \| 466 \| 466; R-squared \| 0.662 \| 0.696; Number of groups \| 31 \| 31; area \| YES \| YES; year \| YES \| YES *These analyses have been incorporated into the revised version of our paper, and we have discussed the implications of the findings in the context of time trend effects. We believe that these additions have significantly enhanced the robustness and comprehensiveness of our study.* ***We sincerely thank you for your guidance and for suggesting the four papers, which we have included in our references, and have been invaluable in refining our methodology.*** |
| ***Results and Analysis***  ***1. Presentation of Findings: Discuss the impact of regional educational policies on UIR collaborative innovation, drawing clear connections between policy variables and innovation outcomes.*** | *Thank you for your constructive feedback on the Results and Analysis section. In response, we have made significant efforts to ensure that the presentation of findings directly addresses your suggestions.*  ***Please refer to Pages 26-28 in the revised manuscript.*** *Below is a summary of how the revised manuscript fulfills your recommendations:*  **1.Clear Connections Between Policy Variables and Innovation Outcomes:**  The added discussion section explicitly discusses how regional education policies have fostered university-industry-research (UIR) collaboration, as evidenced by the increased number of joint patent applications (Table 4). This demonstrates the causal relationship between policy interventions and enhanced innovation outcomes.  To address regional disparities, we analyzed the differential impacts of policies across regions with varying economic foundations (Hypothesis 2a). For example, stronger economic regions like Jiangsu benefit more significantly from these policies, while regions with weaker foundations face challenges due to limited resources.  We also explored how innovation intensity of output influences policy effectiveness (Hypothesis 2b), revealing that regions with high-output intensity experience stronger positive effects, particularly in high-tech industries.  The study identifies key drivers—human capital, government support, economic capital, and social stability—that mediate the relationship between policies and UIR collaboration (Hypothesis 3). For example, human capital emerged as the most critical driver, consistent with prior literature emphasizing the role of skilled talent in fostering innovation.  **2. Global Relevance and Implications:**  The findings are not limited to China; they align with global studies on regional innovation systems, including cases from Germany, Ireland, and Japan. This underscores the broader applicability of regional education policies in fostering collaborative innovation.  *We believe these revisions have strengthened the Results and Analysis section, addressing your suggestions with clear connections, robust analysis of heterogeneity, and actionable insights. Thank you again for your valuable feedback.* |
| ***Results and Analysis***  ***2. Time Series Insights: Present unit root test results to establish the consistency of the data, and interpret the time-fixed effects, explaining how they reflect the evolution of policy impacts over time.*** | *Thank you very much for your valuable comments. In response to your insights regarding the time series analysis, we have conducted a thorough analysis and supplemented the relevant content.* ***Please refer to Page 12-13 and Page 20-21 in the revised manuscript.***  Firstly, we employed the ADF unit root test to determine the stationarity of each variable. The test results indicated that some variables were not stationary in their original form. Therefore, we applied first-difference transformation to these variables and conducted the ADF test again. The results showed that all variables rejected the null hypothesis after first-difference transformation, indicating that they are all first-order stationary sequences, thus ensuring the stationarity and consistency of the data. Furthermore, to verify the existence of a long-term cointegration relationship among the variables, we conducted an EG two-step test on the residuals. The test results revealed that the original sequence of residuals was stationary, confirming the cointegration relationship among the variables.  Regarding the time trend effects, we adopted two methods to analyze and exclude their potential impact. The first method involved including the time trend term as a control variable in the regression model. The results showed that the core explanatory variable (DID) remained significant after excluding the influence of the time trend, indicating that the results of DID were not affected by the time trend. The second method involved interacting DID with multi-year time dummy variables and including the interaction term as a control variable in the regression. The results similarly demonstrated the validity of the DID effect.  *We have presented these analysis results in detail in the paper and interpreted the time-fixed effects, elucidating how they reflect the evolution of policy impacts over time. Thank you again for your review and guidance.* |
| ***Policy Implications***  ***1.Relevant Policy Insights:***  ***Innovation Ecosystem Support: Recommend policies to create supportive environments for UIR collaborations, such as funding incentives and platforms for industry-academia partnerships.***  ***Educational Integration Policies: Encourage enhanced integration of educational institutions into innovation-driven economic frameworks, especially in tech hubs and high-growth regions in China.***  ***Future Directions: Suggest policies that could further regional integration by focusing on digital transformation in educational frameworks, particularly in fields relevant to the emerging needs of the Chinese economy, such as AI and renewable energy*** | *Thank you for your thoughtful feedback on the policy implications section. We have carefully revised this part of the manuscript to incorporate your suggestions and ensure that the proposed policies are both actionable and aligned with the study's findings.* ***Please refer to Pages 28-29 in the revised manuscript.*** *Below are the specific improvements made:*  **1.Innovation Ecosystem Support**  In the revised policy implications, we emphasize the importance of creating supportive environments for university-industry-research (UIR) collaborations. This includes recommending policies such as the establishment of dedicated research funds and innovation funds, particularly targeting high-priority sectors like AI and renewable energy.  We also propose the creation of shared technology platforms and regional innovation hubs to foster collaboration between academia and industry, ensuring the effective integration of resources and enhancing technological advancement. These actionable mechanisms are designed to address the gaps between educational institutions and industrial needs, as highlighted by our findings.  **2.Enhanced Educational Integration Policies**  We have highlighted the critical role of aligning regional education reforms with long-term economic goals, particularly in tech hubs and high-growth regions. The revised section discusses how educational institutions can be integrated into innovation-driven frameworks through initiatives such as joint academic-industrial programs and the development of specialized training programs tailored to the needs of emerging industries.  These efforts aim to improve the quality of human capital and strengthen the linkage between education and industry, ultimately accelerating regional innovation and industrial transformation.  **3.Future Directions: Digital Transformation and Emerging Fields**  Addressing your suggestion on future policy directions, we have proposed that regional education policies prioritize digital transformation, particularly in fields like AI and renewable energy. The revised section outlines strategies for supporting high-risk, high-reward research and disruptive technological exploration in regions with strong innovation ecosystems.  For regions with weaker innovation foundations, the focus is on improving educational infrastructure and basic research capabilities, ensuring a balanced development trajectory across different regions.  *These revisions ensure that the policy implications are comprehensive, actionable, and relevant to both China's emerging economic needs and broader global innovation challenges. Thank you again for your valuable suggestions, which have greatly enhanced the clarity and practical relevance of this section.* |
| ***Conclusion and Future Scope***  ***1. Summarize Contributions and Limitations: Briefly summarize how the study contributes to understanding the effects of regional educational policies on collaborative innovation, while noting any limitations in scope or methodology.*** | *Thank you for your valuable suggestions regarding the Conclusion and Future Scope sections. In response, we have thoroughly revised these sections to address your concerns.* ***Please refer to Pages 28-29 in the revised manuscript.*** *Below are the key points:*  **1.Contributions of the Study:**  The study employs a Difference-in-Differences (DID) model to quantitatively assess the impact of regional education integration policies on UIR collaborative innovation. This contributes to the literature by providing empirical evidence on how these policies enhance innovation through mechanisms such as human capital, government support, economic capital, and social stability.  It also highlights the heterogeneity of policy effects, showing greater impact in regions with stronger economic and educational foundations and on science and technology-driven innovation collaborations.  The findings offer actionable policy insights, emphasizing the integration of regional education reforms into broader innovation-driven development strategies to optimize talent cultivation, resource allocation, and industrial transformation.  **2.Acknowledgment of Limitations:**  We recognize that the data and policy context are primarily focused on specific regions in China, which may limit the generalizability of the findings.  Additionally, the study examines short- and medium-term effects, without considering the long-term impacts of regional education policies in the digital era, particularly their interactions with technological and social innovations.  To address these limitations, we propose expanding the study to include other Asian economies or a global context for cross-national comparisons, which will help validate the findings and uncover broader policy implications.  Furthermore, future research could explore the long-term effects of digital education policies on UIR ecosystems, focusing on innovations such as online learning platforms, digital infrastructure, and AI-driven educational tools. These extensions will provide forward-looking insights for optimizing the synergy between education and innovation policies.  *We hope these revisions effectively address your concerns. Thank you again for your valuable feedback, which has significantly improved the clarity and relevance of this section.* |
| ***Conclusion and Future Scope***  ***2. Future Scope: Propose avenues for further research, such as expanding to other Asian economies or assessing the long-term impact of digital educational policies on UIR collaborations.*** | *Thank you for your insightful feedback regarding the Conclusion and Future Scope section. In response to your comments, we have revised the manuscript to include a detailed discussion of the study's limitations and propose future research directions that align with your suggestions.* ***Please refer to Pages 29 in the revised manuscript.*** *Specifically:*  **1.Regional Expansion:**  Acknowledging the current study’s focus on specific regions in China, we propose expanding the scope to include other Asian economies or even a global context. This extension would enable cross-national comparisons, offering insights into the universality and boundary conditions of our findings. Additionally, such studies would deepen our understanding of how institutional contexts and regulatory mechanisms influence policy outcomes in diverse settings.  **2.Long-Term Impact of Digital Educational Policies:**  We highlight the need for future research to explore the long-term effects of digital educational policies on UIR collaboration ecosystems. Key areas of investigation could include the role of digital teaching, online platforms, and educational infrastructure in shaping innovation outputs, technology diffusion, and industrial upgrading. These studies would provide forward-looking insights into how education and innovation policies can synergize to address emerging challenges in a dynamic global landscape.  *We believe these additions strengthen the manuscript by providing a forward-looking perspective and addressing the broader implications of our findings. Thank you again for your constructive suggestions, which have greatly improved the clarity and relevance of this section.* |
| ***Proofreading and Language Precision***  ***Grammar and Typing Checks: Ensure that the document is thoroughly proofread for typing errors, grammatical issues, and readability. Utilize tools like Grammarly or Hemingway to catch minor errors and ensure that the language is concise and academic.*** | *Thank you for your valuable feedback. We have thoroughly proofread the manuscript to ensure it is free from typing errors, grammatical issues, and readability concerns. In response to your suggestion, we have utilized tools like Grammarly and Hemingway to identify and correct minor errors and ensure that the language is concise, clear, and academic.*  *We have carefully revised the manuscript to enhance its precision and readability, addressing any issues that may have affected the overall flow and comprehension. We believe these revisions have improved the quality of the manuscript and have made it more polished and professional.*  *Thank you again for your helpful comments.* |
