# Supplementary material for: Assessing the role of regional educational integration policies in fostering university-industry-research innovation: Evidence from China
Source: PLoS One. 2025 Jul 18;20(7):e0325717. doi: 10.1371/journal.pone.0325717 (PMC12273934; doi:10.1371/journal.pone.0325717)
Supplement: Attachment — Submitted filename: 125.docx [file pone.0325717.s005.docx]

**Review Report on Manuscript Titled: "Assessing the Role of Regional Education Integration Policies in Fostering University-Industry-Research Innovation: Evidence from China"**

**Evaluation Summary:** The manuscript provides an insightful and well-structured analysis of the impact of regional education integration policies on fostering university-industry-research (UIR) collaboration and innovation in China. The authors have thoroughly addressed previous comments raised by reviewers, resulting in significant improvements in the clarity, depth, and overall quality of the manuscript. Based on my observations, the authors have successfully met all requirements.
