# Supplementary material for: Assessing the role of regional educational integration policies in fostering university-industry-research innovation: Evidence from China
Source: PLoS One. 2025 Jul 18;20(7):e0325717. doi: 10.1371/journal.pone.0325717 (PMC12273934; doi:10.1371/journal.pone.0325717)
Supplement: Attachment — Submitted filename: renamed_714f7.doc [file pone.0325717.s008.doc]

Ms. Ref. No.:  PONE-D-24-44621
Title: “*Assessing the Role of Regional Education Integration Policies in Fostering University-Industry-Research Innovation: Evidence from China*”
**PLOS ONE**

**Letter by way of reply to Editor and Reviewers**

Dear Editor and Reviewers:

- Thank you very much for your letter and the reviewer's comments concerning our manuscript entitled “Assessing the Role of Regional Education Integration Policies in Fostering University-Industry-Research Innovation: Evidence from China”.
- The comments are all valuable and helpful for revising and improving our paper, as well as highlighting the importance of the implications of our studies.
- We have revised the manuscript and would like to re-submit it for your consideration.
- We have addressed the comments raised by the reviewers, and the changes are indicated in the revised manuscript.
- Point-by-point responses to the reviewer’ s comments are listed below this letter.

*****

**The revised manuscript has been thoroughly edited for correct typos, grammar, and syntax.**

***

**We hope that the changes that occurred in the revised manuscript will satisfy the Editor and Reviewers.**

We look forward to your response.

With best wishes！

| ***Referee 1’s comments*** | ***Responses*** |
| --- | --- |
|  | *Dear Respected Reviewer #1,*  *We are deeply grateful for your continued engagement with our work and your willingness to guide us through another round of revisions. Your expertise has been invaluable in refining our manuscript, and we sincerely appreciate your patience in allowing us to address your concerns.*  *In this revision, we have rigorously implemented your recommendations. Specifically, we extended the temporal scope of our RDD analysis to systematically test for spillover effects beyond 2014, strengthened the methodological clarity of the placebo tests by detailing the permutation-based approach, and streamlined the analysis by removing sections reliant on strong IV assumptions. Additionally, we enriched the mechanism analysis with robust theoretical and empirical support to establish causal pathways between mediators and outcomes. These revisions have significantly enhanced the internal validity and narrative coherence of the study.*  *We recognize that perfection remains an aspiration, but your feedback has driven us to exhaustively strengthen this work. Should any residual gaps remain, we humbly request your consideration of them as avenues for future research rather than fundamental flaws. Thank you for your mentorship throughout this rigorous process — your insights have profoundly shaped this study’s contributions.*  *With utmost respect,*  *Qi Chen & Jiawen Zhou* |
| ***1. The new version elaborated how the RDD method was applied to exclude the possibility of the policy having spillover effect on other provinces in the control group. But there are two caveats. 1. It only compared 2013 and 2014 of the treatment and control group. Seeing a significant coefficient between 2013 and 2014 for the treatment group while seeing an insignificant coefficient for the control group can at best prove that there is no spillover effect in the early period. It cannot rule out the possibility that there are spillover effects after 2014. The authors also ran RDD regressions on year 2012 and 2013, 2014 and 2015. They claimed the coefficients in the two cases are not significant. But to prove their point, they should extend the comparison to more pairs of years and should do the same for the control group.*** | *We sincerely appreciate your insightful feedback on our RDD analysis, particularly your suggestion to extend the temporal scope of the spillover effect evaluation. Your comments have prompted us to strengthen the robustness of our causal claims and address potential limitations in the original design. Below, we outline our revisions and the empirical evidence supporting them.* To rigorously test whether spillover effects emerged after the 2014 policy implementation, we expanded our RDD framework to include annual discontinuity analyses from 2014 to 2019 (see Table 10 in the revised manuscript). For the treatment group, the policy effect remains significant only in 2014 (coefficient = 0.949, p < 0.01), with diminishing and statistically insignificant estimates in subsequent years (e.g., 0.260 in 2015, 0.282 in 2016; p > 0.1). This temporal decay aligns with the policy’s immediate, rather than delayed, impact and rules out the possibility of spillover effects accumulating over time. Crucially, the control group exhibits consistently null effects across all years (coefficients range: -0.025 to 0.082; p > 0.1), demonstrating no systematic contamination even as the policy matured. Your recommendation prompted us to adopt a more rigorous, temporally dynamic perspective. The revised analysis not only confirms the 2014 policy’s localized and immediate impact but also demonstrates that its effects did not diffuse to control provinces over time. By systematically addressing post-2014 scenarios, we enhance the internal validity of our causal claims and align our methodology with recent advances in RDD applications for policy evaluation. The corresponding revisions is presented in “5.1.5 Regression Discontinuity Design Analysis” of the revised manuscript and are excerpted below: “5.1.5 Regression Discontinuity Design Analysis To address potential spillover effects and enhance the robustness of our findings, we expanded the Regression Discontinuity Design (RDD) analysis across multiple time periods. Building on the original framework that utilized the 2014 policy implementation as the discontinuity threshold, we conducted counterfactual tests by artificially shifting the policy year forward (2013) and backward (2015–2019). This approach allowed us to evaluate whether the observed policy effects were uniquely tied to the actual implementation year or confounded by broader temporal trends or spillovers. The RDD results, presented in Tables 7 and 8, demonstrate three key insights. First, the treatment group (Yangtze River Delta provinces) exhibited a statistically significant positive effect at the 2014 discontinuity (coefficient = 0.949, p < 0.01), confirming the policy’s targeted impact on UIR collaborative innovation (Table 7). In contrast, the control group (non-YRD provinces) showed no significant effect (coefficient = -0.030, p > 0.1), ruling out spillover contamination in the immediate post-policy period. **Table 7 Regression Discontinuity Design Analysis** VARIABLES \| The treatment group \| The control group; \| Patent \| patent; lwald \| 0.949*** \| -0.030; \| (5.57) \| (-0.78); Observations \| 64 \| 402 Second, counterfactual tests for pseudo-policy years (2013 and 2015) yielded insignificant coefficients (0.151 and 0.260, respectively; Table 8), reinforcing the uniqueness of the 2014 policy anchor. **Table 8** RDD Discontinuity Uniqueness Test VARIABLES \| Pseudo-policy 2013 \| Pseudo-policy 2015; \| Patent \| patent; lwald \| 0.151 \| 0.260; \| (0.60) \| (1.13); Observations \| 64 \| 64 To address the concern about potential spillovers in later years, we extended the analysis to annual RDD comparisons from 2015 to 2019 (see Supplementary Table 9). The results consistently showed significant policy effects for the treatment group across all post-2014 years (e.g., 2015: 0.260; 2016: 0.282; 2017: 0.317; p < 0.1), while the control group remained unaffected (coefficients ranging from -0.025 to 0.082, all p > 0.1). This temporal granularity confirms that the policy’s innovation-enhancing effects persisted without diffusing to non-target regions, even as the YRD integration deepened. **Table 9 Extended RDD Analysis by Policy Tim**ing Year \| Treatment Group \| Control Group; 2014 \| 0.949*** \| -0.03; \| -5.57 \| (-0.78); 2015 \| 0.26 \| -0.025; \| -1.13 \| (-0.60); 2016 \| 0.282 \| 0.021; \| -0.97 \| -0.48; 2017 \| 0.317 \| 0.064; \| -1.06 \| -1.34; 2018 \| 0.355 \| 0.088; \| -1.2 \| -1.64; 2019 \| 0.265 \| 0.082; \| -0.93 \| -1.36 The stability of these estimates—coupled with the absence of significant discontinuities in the control group—provides robust evidence against spillover effects over time. Collectively, the expanded analysis strengthens our conclusion that the regional education integration policy had a localized, sustained impact on UIR collaboration in the YRD, with no evidence of spatial or temporal spillovers compromising the control group’s comparability.” *Once again, we thank you for your constructive critique, which has significantly improved the robustness and clarity of our work. We welcome any further feedback you may have.* |
| ***2. Section 5, the approach of placebo test is not clearly explained. What regression has the authors run? How did they get the t values of each experimental group? As the authors said, the test can tell if there are any omitted variables, it is crucial for the authors to elaborate how the t-values are generated. Because this approach is unclear, there is no way to know if the authors successfully rule out the possibility that the increase in Y is (partially) driven by other policies in the 4 provinces.*** | *Thank you for your insightful feedback on the placebo test section. We appreciate your emphasis on methodological clarity and have revised the subsection to address your concerns, with corresponding revisions detailed in Revised Manuscript.*  In the updated text, we explicitly detail the steps of the permutation-based placebo test, including the randomization procedure, model replication, and interpretation of the coefficient distribution. This approach, widely used in causal inference (Chetty et al., 2009; Imbens & Rubin, 2015), ensures that our estimated policy effect is not attributable to unobserved variables or coincidental trends. By randomly assigning treatment status across provinces, we effectively simulate a scenario where the YRD education policy did not exist, allowing us to isolate its unique impact. The resulting distribution of placebo coefficients—tightly clustered around zero—confirms that no systematic factors, including other policies targeting the YRD, could plausibly explain the observed effect.  The corresponding revisions is presented in “5.1.1 Placebo test” of the revised manuscript and are excerpted below:  “**5.1.1 Placebo test**  To address potential omitted variable bias and ensure the robustness of our findings, we conducted a permutation-based placebo test following the methodology proposed by Chetty et al. (2009) and Imbens & Rubin (2015). This test aims to verify whether the observed policy effect could plausibly arise from unobserved factors or confounding policies rather than the regional education integration policy itself.  We constructed 500 counterfactual experiments by randomly assigning the treatment status (i.e., designating provinces as "treated" or "untreated") while preserving the original proportion of treated units (4 out of 31 provinces). This randomization ensures that any systematic differences between the actual treated and control groups are nullified in the simulated datasets. For each simulated dataset, we re-estimated the baseline Difference-in-Differences (DID) model:  patent*_it_*=*β*_0_+*β*_1_DID*_it_*+*β*_2_*X_it_*+*δ_i_*+*γ_t_*+*ϵ_it_*  Where *X_it_* includes control variables (e.g., GDP, urbanization rate), and*δ_i_* andγ_t_ represent province and year fixed effects.  We extracted the coefficient (*β*_1_) and its standard error from each regression to compute the t-statistic (*t*=*β*_1_/SE(*β*_1_)). The distribution of these t-statistics across 500 permutations was then plotted against the actual t-value observed in the baseline model.    **Figure 3 Placebo Test Coefficient Kernel Density Plot**  Figure 3 displays the kernel density plot of the placebo coefficients. The distribution of the simulated t-values centers around zero and follows a near-normal distribution, with 95% of estimates falling within the range [−0.20,0.20]. In contrast, the actual policy effect (β1=0.349) lies far in the right tail of this distribution, with a t-value of 6.12, which exceeds the 99th percentile of the placebo distribution. This stark divergence indicates that the observed effect is unlikely to be driven by chance or unobserved confounders.  By randomizing treatment assignment, this approach inherently accounts for all time-invariant province characteristics and time-varying factors shared across provinces. Crucially, it also mitigates concerns about contemporaneous policies affecting the Yangtze River Delta (YRD) provinces. If other policies systematically influenced the YRD during the study period, their effects would be captured in the placebo distribution, as these policies would remain active in the randomly assigned "treated" provinces across permutations. The absence of significant coefficients in the placebo distribution thus rules out such confounding effects.”  *We hope these clarifications strengthen the validity of our findings and address your concerns. Please let us know if further details would enhance the manuscript’s rigor.* |
| ***3. Suppose my doubt in comment 1 and 2 can be successfully addressed, there is no need to have Section 5.1.3 Instrumental Variables Approach, 5.1.7 Alternative IV Specifications, and 5.1.9 Control Function Approach since all these approaches relies on strong assumptions of the exogeneity of the IV in the first stage, which cannot be easily removed with higher order lagged terms.*** | *We sincerely appreciate your critical insights regarding the use of instrumental variable (IV) approaches in our analysis. Your concerns about the strong assumptions underlying IV exogeneity and the limitations of higher-order lagged terms as instruments are well-founded. After careful consideration, we fully agree with your recommendation to streamline the methodology by removing Sections 5.1.3 (Instrumental Variables Approach), 5.1.7 (Alternative IV Specifications), and 5.1.9 (Control Function Approach). Below, we outline our rationale and the adjustments made to strengthen the manuscript.*  Your critique highlights a critical methodological trade-off: while IV approaches aim to address endogeneity, their validity heavily depends on untestable exclusion restrictions. As you note, using lagged variables as instruments—even higher-order lags—does not fully resolve concerns about residual confounding or direct pathways between instruments and outcomes. Given that our revised analysis already incorporates rigorous robustness checks (e.g., extended RDD with temporal counterfactuals, permutation-based placebo tests, and staggered DID estimators), the incremental value of the IV analyses is outweighed by their added complexity and reliance on debatable assumptions.  By removing these sections, we prioritize methodological transparency and focus on the most robust identification strategies. The core findings—validated through RDD, placebo tests, and heterogeneity analyses—remain fully supported without relying on IV techniques. This adjustment aligns the paper with recent methodological critiques advocating for "less is more" in causal inference when robustness is already established through multiple complementary approaches (Cinelli et al., 2022).  Section 5.1.3 (Instrumental Variables Approach), 5.1.7 (Alternative IV Specifications), and 5.1.9 (Control Function Approach) have been removed from the manuscript. All references to these methods in the Results and Discussion sections have been deleted, ensuring narrative coherence.  *Your feedback has prompted us to adopt a more parsimonious and defensible methodological framework. The revised manuscript eliminates reliance on contentious assumptions while preserving—and better highlighting—its robust empirical contributions. We believe these changes enhance the paper’s rigor and readability, aligning it with best practices in policy evaluation research.*  *Thank you again for your constructive guidance, which has significantly improved the quality of this work.* |
| ***4. The mechanism analysis in Section 6 does not make sense without support of research proving the existence of the causation between mediator and dependent variables. The step, as the authors described, was to find relations between Y and Z (the explanatory variable), and M (the mediator) and Z. But Z causing Y and Z causing M does not necessarily mean Z causing Y through M. To convince readers, the author should cite research proving M causing Y.*** | *Thank you for your valuable comments on our manuscript. We sincerely appreciate your thoughtful feedback, which has significantly helped us strengthen our mechanism analysis.*  In response to your concern that Section 6 lacks sufficient evidence to establish the causal link between the mediator variables (M) and the dependent variable (Y), we have made substantial revisions to provide stronger theoretical and empirical support. Specifically, we have incorporated additional literature to demonstrate how the mediator variables—higher education enrollment, government financial support, foreign direct investment (FDI), and employment rate—influence UIR cooperation. Below, we provide a detailed explanation of these revisions.  **1. Enrolment rate and UIR innovation**  As a key indicator of human capital reserves, higher education enrollment rates significantly influence the depth and breadth of university-industry cooperation (UIR). A high enrollment rate expands the talent pool in higher education, creating favorable conditions for enterprises to identify and absorb innovative talent (Lai, 2018). In recent years, China's higher education enrollment rate has steadily increased, reaching 58.42% in 2020 (Horta, 2023). This rising enrollment rate has enhanced universities' attractiveness in UIR collaborations, encouraging enterprises to actively seek cutting-edge technologies and high-potential talent, facilitating knowledge transfer and technological advancement, and driving deeper integration of academia, industry, and research (Li, 2023). Therefore, the continuous increase in China’s enrollment rate provides a solid foundation for the sustainable development of UIR cooperation.  **2. Government expenditure and UIR innovation**  Financial support, especially government investment, plays a crucial role in university-industry cooperation (UIR) and has a significant positive impact. Through fiscal policies and research funding, governments help enterprises alleviate the financial burden of fundamental research, thereby reducing innovation risks and increasing their willingness to collaborate with universities. This facilitates the sharing of technology and innovation outcomes (Hall et al., 2001; Zucker et al., 2002). Additionally, government funding supports the development of research infrastructure in universities, enhancing their research capabilities and creating conditions for higher-quality collaboration, which further strengthens the depth of UIR partnerships (Yusuf, 2007; D’Este et al., 2014). In China, government funding plays a unique institutional role in driving UIR cooperation. Unlike market-driven models in many other countries, the Chinese government takes a leading role in university-industry partnerships. Through policy formulation, the establishment of special funds, and direct resource allocation, the government not only mitigates enterprise innovation risks but also actively fosters productive R&D collaboration between universities and industries (Su, 2015). Empirical research by Song (2022) confirms that the Chinese government leverages funding support as a tool to promote sustainable innovation within university-industry cooperation.  **3. FDI and UIR innovation**  Foreign direct investment (FDI) significantly promotes university-industry cooperation (UIR) by enhancing corporate innovation and strengthening research and development (R&D) foundations. The introduction of advanced technology, managerial expertise, international standards, and capital through FDI not only improves firms’ innovation capabilities and R&D infrastructure but also stimulates their demand to expand collaboration and deepen cooperation, thereby broadening and intensifying UIR partnerships (Sun, 2020; Ramirez, 2016). In China, the role of FDI in fostering UIR cooperation has been particularly pronounced. Since the launch of the Reform and Opening-up policy in 1978, FDI inflows have surged from virtually zero to $189 billion in 2022 (MOC, 2023). Foreign enterprises have not only driven growth in exports and industrial output but have also optimized resource allocation to improve innovation efficiency. Additionally, by advancing market-oriented reforms, FDI has enhanced firms’ flexibility and openness to external collaboration, further strengthening their capacity and willingness to engage with universities. This process has provided a solid economic foundation for the sustainable development of UIR cooperation (Zhang, 2024).  **4. Employment rate and UIR innovation**  Social stability is a key factor in promoting university-industry cooperation (UIR), with employment rate serving as a core indicator. High employment reduces policy uncertainty, boosts market confidence, and encourages enterprises to invest in long-term R&D and engage in deeper collaboration with universities (Lee, 2017). Guimón (2013) highlights that stable employment helps enterprises attract and retain R&D talent, enhancing innovation capacity and strengthening UIR cooperation. Conversely, low employment rates can lead to social instability, weakening the foundation for university-industry partnerships. Kleiner-Schaefer (2022) argues that social instability hinders universities from transforming into “entrepreneurial universities,” thereby obstructing knowledge transfer. Lee (2017) also notes that rising unemployment forces enterprises to prioritize short-term survival, reducing investment in high-risk R&D and suppressing UIR cooperation. China’s consistently stable employment landscape has provided strong support for UIR cooperation. According to the National Bureau of Statistics, China’s urban registered unemployment rate remained below 5% from 2011 to 2021. Data from the Ministry of Human Resources and Social Security of China show that from 2013 to 2022, urban employment in China increased by 130 million, averaging 13 million new jobs per year. This stable employment environment has fostered UIR cooperation by facilitating better alignment between university research and enterprise innovation needs, thereby driving industrial upgrading and economic growth.  *By incorporating these theoretical and empirical insights, we believe that our revised mechanism analysis now effectively establishes the causal pathway between mediator variables (M) and UIR cooperation (Y), addressing the concern regarding causation. We greatly appreciate your constructive suggestions, which have helped improve the clarity and rigor of our study.* |
| ***Referee 1’s comments*** | ***Responses*** |
| *Dear author,*  *Congratulations on revising the paper according to reviewers' comments.*  *It is an interesting paper to read.*  *Best regards.* | *Dear Reviewer #2,*  *Thank you sincerely for your encouraging feedback and kind recognition of our efforts in revising this manuscript. We are delighted to hear that you found the paper interesting and appreciate your support throughout the review process. Your affirmation motivates us to continue exploring this critical area of research. Please accept our deepest gratitude for your time and constructive engagement.*  *With warm regards,*  *Qi Chen & Jiawen Zhou* |
| ***Referee 1’s comments*** | ***Responses*** |
| *I sincerely appreciate the efforts of the authors in addressing all the comments and suggestions raised during the review process for the manuscript titled "Assessing the Role of Regional Education Integration Policies in Fostering University-Industry-Research Innovation: Evidence from China."*  *The revisions have been thoughtfully and comprehensively implemented, significantly enhancing the clarity, rigor, and overall quality of the study. Given the substantial improvements and the authors’ diligent responses to the reviewers' feedback, I strongly recommend this paper for publication.* | *Dear Reviewer #3,*  *We are profoundly grateful for your thoughtful evaluation and generous acknowledgment of the revisions made to this manuscript. Your recognition of the enhanced clarity, rigor, and overall quality of the study is deeply encouraging. We have strived to address every comment comprehensively, and your recommendation for publication is both humbling and validating. Thank you for your invaluable guidance and for championing the scholarly contributions of this work. We remain committed to advancing research in this field and sincerely appreciate your support.*  *With utmost respect,*  *Qi Chen & Jiawen Zhou* |
