# Supplementary material for: Assessing the role of regional educational integration policies in fostering university-industry-research innovation: Evidence from China
Source: PLoS One. 2025 Jul 18;20(7):e0325717. doi: 10.1371/journal.pone.0325717 (PMC12273934; doi:10.1371/journal.pone.0325717)
Supplement: Attachment — Submitted filename: 20250505 Response to Reviewers.doc [file pone.0325717.s009.doc]

Ms. Ref. No.:  PONE-D-24-44621
Title: “*Assessing the Role of Regional Educational Integration Policies in Fostering University-Industry-Research Innovation: Evidence from China*”
**PLOS ONE**

**Letter by way of reply to Editor and Reviewers**

Dear Editor and Reviewers:

- Thank you very much for your letter and the reviewer's comments concerning our manuscript entitled “Can regional educational integration policy promoted university- industry-research collaborative innovation? –Evidence from China”.
- The comments are all valuable and helpful for revising and improving our paper, as well as highlighting the importance of the implications of our studies.
- We have revised the manuscript and would like to re-submit it for your consideration.
- We have addressed the comments raised by the reviewers, and the changes are indicated in the revised manuscript.
- Point-by-point responses to the reviewer’ s comments are listed below this letter.

*****

**The revised manuscript has been thoroughly edited for correct typos, grammar, and syntax.**

***

**We hope that the changes that occurred in the revised manuscript will satisfy the Editor and Reviewers.**

We look forward to your response.

With best wishes！

| ***Referee #1’s comments*** | ***Responses*** |
| --- | --- |
| ***The author has used multiple methods to address the concern of potential endogeneity. One of the concern comes from spillover effect. The author regress Y on treatment and control groups' post treatment period, respectively, and found the significant correlation only existed in the treatment group. This is a strong evidence to rule out spillover effect. The author attempted to address the concern of simultaneous effects by randomly assign hypothetical treatment to other groups, which can help convince readers but cannot fully address the issue. The simultaneous effects is due to the unique position of the treated regions in China's economy, which the placebo test cannot capture. Although the concern has not been fully addressed, I am impressed by the authors' efforts to make improvement according to my comments. Thus, I would give this quite polished paper a pass.*** | *Dear Respected Reviewer #1,*  *We are deeply grateful for your unwavering support and meticulous guidance throughout the revision process of this paper. Your expertise, patience, and constructive feedback have been invaluable in shaping the manuscript into its current form.*  *Your recognition of the efforts to address endogeneity concerns, particularly the spillover effect analysis and placebo tests, is immensely encouraging. While we fully acknowledge that the simultaneous effects related to the treated regions’unique economic positions remain a limitation—as you rightly pointed out—your insightful critique has not only strengthened this paper but also deepened our understanding of methodological rigor in empirical research.*  *Please know that your dedication to nurturing early-career scholars like ourself is profoundly appreciated. Even as we recognize the imperfections in this work, we will carry forward the lessons learned from your feedback into our future research. Your emphasis on robust identification strategies and nuanced interpretation of results has already become a cornerstone of our academic practice.*  *Once again, thank you for investing your time and wisdom in improving this study. Your generosity in balancing rigorous standards with genuine support for scholarly growth embodies the very best of academic mentorship. We will strive to pay forward the confidence you have placed in us.*  *With utmost respect,*  *Qi Chen & Jiawen Zhou* |

| ***Reviewer #3’s comments*** | ***Responses*** |
| --- | --- |
| ***1. Title Analysis: The title reflects an investigation into the role of regional educational integration policies in promoting collaborative innovation between universities, industries, and research institutions. However, it can benefit from a slightly refined structure to highlight the relationship between policy integration and innovation outcomes more clearly. Consider rephrasing to, “Assessing the Role of Regional Educational Integration Policies in Fostering University-Industry-Research Innovation: Evidence from China.”*** | *Thank you for your valuable suggestion regarding the title. In response to your recommendation, we have revised the title to: “****Assessing the Role of Regional Educational Integration Policies in Fostering University-Industry-Research Innovation: Evidence from China****” .*  *Thank you for your insightful suggestion.* |
| ***2. Coherence Comment: In assessing the manuscript, ensure the logical flow between sections is clear, linking regional education policies directly with mechanisms that support collaboration across universities, industries, and research entities. For instance, demonstrate how specific policies lead to actionable frameworks or platforms for innovation.***  ***Introduction***  ***1. Introduce Latest Literature: Begin with a strong theoretical framework around regional education integration and collaborative innovation, connecting it with recent literature.*** | *Thank you very much for your valuable feedback and insightful suggestions on our manuscript. We have significantly enhanced the theoretical framework around regional education integration and collaborative innovation. We now begin with a strong foundation in three core theories: The Growth Pole Theory, Synergetics Theory, and Innovation Theory, which together provide a robust framework for analyzing the role of policies in fostering regional innovation.*  *To connect with recent literature, we have incorporated several recent studies to highlight the relevance of these theories in understanding the dynamics of regional education integration and innovation. Specifically in the revised manuscript page 2.*  **1. Growth Pole Theory emphasizes the role of economic core areas as hubs for innovation and resource concentration**, which can drive development in surrounding regions. Studies show that higher education quality is a key to human capital and technological innovation, driving regional economic growth [6]. Zhang [7] applies this theory to explain the relationship between higher education and regional innovation, emphasizing the agglomeration effect in enhancing innovation capacity.  **2. Synergetics Theory highlights the importance of coordination among diverse innovation actors**, such as universities, industries, and governments, in achieving dynamic equilibrium and fostering collaborative innovation. Jafarov [8] uses this framework to explore how collaboration between education and innovation elements optimizes regional development. Wang et al. [9] further apply this theory to analyze the interaction between higher education and regional innovation, with a focus on regional economic and innovation growth.  **3. Innovation Theory focuses on the mechanisms through which policies stimulate technological progress and enhance regional innovation capacity.** Li et al. [10] highlight the central role of education in regional innovation and its interaction with innovation and digitalization, aligning with core concepts of "human capital" and "innovation ecosystems." Qing et al. [11] utilize Innovation Theory to explore the impact of regional policies, resource allocation, and human capital on innovation efficiency.  *These references help to ground our study in existing research and clarify the theoretical underpinnings for understanding the relationship between regional education policies and collaborative innovation.*  *These revisions have strengthened the logical coherence of the paper and better articulated the theoretical framework in relation to recent literature.*  *Thank you once again for your insightful comments.* |
| ***Introduction***  ***2. Enhance Background Context: Emphasize the significance of collaborative innovation in the Chinese economy and regional policy environment, detailing how education integration is aligned with national innovation goals. Highlight the gap your study addresses in bridging these sectors through structured policies.*** | *Thank you for your thoughtful and valuable feedback on our paper. We have made the necessary revisions to address your suggestions as followings:*  **1. Emphasizing the significance of collaborative innovation in the Chinese economy:** we have added the following sentence after the second paragraph in the revised manuscript page 3.  “Collaborative innovation has become a vital component in advancing national economies, particularly in China, where it is central to transitioning towards a more innovation-driven economy.”  This addition highlights the importance of collaborative innovation in China’s economic development, particularly its role in driving industrial upgrading and enhancing global competitiveness.  **2. Aligning education integration with national innovation goals:**  We have also included the following sentence to emphasize how education integration aligns with China’s national innovation agenda in the revised manuscript page 3.  “The integration of regional education systems plays a key role in aligning educational advancements with the nation's broader innovation agenda, which focuses on fostering technological advancement, industrial upgrades, and global competitiveness.”  This modification clarifies how regional education policies are integral to achieving national innovation goals in China.  **3. Highlighting the gap addressed by our study:**  To more clearly reflect the gap our study addresses, we have added the following sentence in the revised manuscript page 3.  “This study addresses this gap by quantitatively assessing the impact of regional education integration policies on UIR collaborative innovation, focusing on regional disparities and the different levels of innovation intensity across regions.”  This addition highlights how our study fills a gap in the existing literature by quantitatively assessing the impact of regional education policies on UIR collaborative innovation, particularly considering regional disparities and varying innovation intensities.  *We believe these revisions strengthen the background context and make the argument of our paper clearer.*  *Thank you once again for your insightful suggestions.* |
| ***Literature Review***  ***1. Summarize Recent Findings: Using the cited studies, build a foundation that captures the scope of recent collaborative models and the role of educational policy in China.***  ***o Key Themes: Mention themes like the role of educational policy in supporting innovation ecosystems, the influence of economic and technological policies, and cross-sectoral partnerships in fostering sustainable innovation.*** | *Thank you for your valuable feedback and insightful suggestions. In response to your suggestion to summarize recent findings and address the key themes in the literature, we have added a concluding paragraph, which explicitly summarizes the three key themes you mentioned in the revised manuscript page 7:*  “Recent findings in the literature increasingly underscore the pivotal role of educational policy in shaping regional innovation ecosystems in China. In particular, studies highlight how education policies are being leveraged not only to cultivate high-level talent and academic excellence, but also to promote industry-oriented research and cross-sectoral collaboration. These developments align with a broader global trend in which regional innovation is no longer driven solely by science and technology policies, but increasingly depends on the integration of education, economic, and industrial strategies. Within China, especially in regions like the Yangtze River Delta, emerging collaborative models show how universities and enterprises are engaging more systematically in knowledge transfer, technology commercialization, and joint R&D, all facilitated by supportive regional education integration policies. These insights provide an analytical foundation for understanding how policies are translated into practice in specific contexts such as industrial parks, university-enterprise alliances, and innovation platforms.”  *This summary connects the findings from the cited studies and highlights how educational policies can support innovation ecosystems, align with regional industrial needs, and promote collaboration between universities, industries, and research institutions.*  *Thank you again for your helpful comments.* |
| ***Literature Review***  ***2. Identify Gaps: Make a clear case for how your paper fills a gap in the existing literature by focusing on China’s unique regional education policy dynamics and their impact on university-industry-research (UIR) collaborations.*** | *Thank you very much for your constructive suggestion. We fully agree that a clear articulation of the research gap and the study’s contribution is essential to enhance the clarity and academic value of the manuscript.*  *To address your comment, we revised Section 2.4 Summary by refining the structure and wording to better highlight the existing literature gaps and our study’s contributions. The revision integrates three previously separate points: (1) the lack of quantitative research on UIR collaboration outcomes; (2) limited analysis of internal mechanisms and regional heterogeneity; and (3) insufficient attention to the Chinese context as a rapidly developing economy with distinct education policy dynamics.*  *We also emphasized how our study fills these gaps by applying a Difference-in-Differences (DID) model to assess the impact of regional education integration policies in China’s Yangtze River Delta region, while also exploring mediating mechanisms and providing insights with potential international relevance.*  *The Revised paragraph is as follows (Section 2.4 Summary in the revised manuscript page 8):*  “Although existing research acknowledges the influence of regional education policies on collaborative innovation, several gaps remain. Most notably, few studies adopt quantitative approaches to directly measure the outcomes of university-industry-research (UIR) cooperation. In addition, the dynamic interactions among collaborating regions and the mediating role of endogenous factors are often overlooked. While regional heterogeneity is sometimes mentioned, it is rarely explored in depth, especially in terms of how economic foundations, educational capacities, and policy execution vary across regions. Moreover, the majority of current research centers on developed countries, with limited examination of China’s unique regional education policy dynamics under its fast-changing socio-economic conditions.  This study addresses these gaps by focusing on the Yangtze River Delta—a leading innovation hub in China—and applying a Difference-in-Differences (DID) model to quantitatively assess the impact of regional education integration policies on UIR collaborative innovation. The analysis considers patent co-application data among universities, enterprises, and research institutions, explores heterogeneous policy effects, and identifies internal mechanisms that mediate education policy outcomes. By grounding its analysis in the Chinese context, this paper contributes to a more nuanced understanding of how education policies function in emerging economies, offering empirical insights with international relevance.”  *We sincerely appreciate your insightful feedback, which helped us significantly improve the coherence and depth of the summary section.* |
| ***Methodology***  ***1. Incorporate Unit Root Tests and Time Effects: For methodological enhancement, integrate unit root tests to assess the stationarity of variables, which is essential for panel data involving time series to ensure robust estimation.***  ***o Suggested Approach: Use LLC, IPS, ADF-Fisher, or PP-Fisher tests to confirm stationarity of data, especially if dealing with longitudinal data on policy impacts and innovation metrics.*** | *We sincerely appreciate your insightful feedback and constructive suggestions for enhancing the methodological rigor of our study. Your recommendations to incorporate unit root tests and explicitly account for time effects have significantly strengthened the robustness of our empirical analysis.*  We sincerely appreciate your insightful suggestions on enhancing the methodological rigor of our study. In response to your recommendation, we conducted comprehensive unit root tests to assess the stationarity of our panel data. Specifically, we employed the Augmented Dickey-Fuller (ADF) test, which is widely recognized in econometric literature for its robustness in handling panel data with time series properties (see Section 4.1, "Variable Stationarity"). The ADF test results (Table 2) confirmed that all first-differenced variables rejected the null hypothesis of non-stationarity at the 1% significance level, ensuring the validity of our regression models. While alternative tests such as LLC and IPS are valuable, the ADF test was selected due to its suitability for our dataset and its alignment with established practices in similar studies on regional innovation policies (e.g., Kumar et al., 2024; Moridian et al., 2024). Additionally, we performed the Engle-Granger cointegration test (Table 3) to verify long-term equilibrium relationships among variables, further strengthening the reliability of our findings.  *Thank you once again for your constructive feedback.* |
| ***Methodology***  ***2. Time Effects Consideration: Recognize time-fixed effects to account for temporal factors that might influence UIR collaborations (e.g., policy shifts, economic events). This will strengthen the robustness of your results by capturing latent trends over time.***  ***Suggested Sources: https://doi.org/10.1007/s10668-023-04391-7, https://doi.org/10.1016/j.heliyon.2024.e33519,https://doi.org/10.1556/032.2024.00017, https://doi.org/10.1016/j.egyr.2024.08.052*** | *Thank you for emphasizing the importance of accounting for temporal factors.*  We fully integrated time-fixed effects into our Difference-in-Differences (DID) model to control for unobserved time-varying confounders, such as macroeconomic fluctuations or nationwide policy shifts. The model specification explicitly includes year fixed effects (γ_t_), as detailed in Equation (1) of Section 3.1. This approach effectively isolates the policy impact from broader temporal trends, as evidenced by the parallel trend test (Figure 2) and the robustness checks excluding COVID-19 impacts (Table 5). Furthermore, we expanded our analysis by interacting the policy variable with yearly dummies (Table 10), confirming that the policy’s dynamic effects align with theoretical expectations. These adjustments align with recent advancements in DID methodology (Callaway & Sant’Anna, 2021) and address potential biases arising from time-dependent heterogeneity.  *Thank you again for your meticulous review. Your expertise has been invaluable in elevating the analytical rigor and interpretative clarity of our work.* |
| ***Results and Analysis***  ***1. Presentation of Findings: Discuss the impact of regional educational policies on UIR collaborative innovation, drawing clear connections between policy variables and innovation outcomes.*** | *Thank you very much for your thoughtful comments and valuable suggestions on our manuscript. We have carefully addressed your recommendation regarding the need to identify gaps in the literature and to more clearly demonstrate how my paper fills these gaps, particularly by focusing on China’s unique regional education policy dynamics and their impact on university-industry-research (UIR) collaborations. We have made several key revisions in the Discussion section to more explicitly highlight the relationship between regional education policies and UIR collaboration outcomes as followings in the revised manuscript page 29-32.*  **1. Policy Variables and Innovation Outcomes:** We have elaborated on how specific policy variables, such as R&D funding and technology transfer encouragement, contribute to the tangible outcomes of UIR collaboration. This is now clearly reflected in the text, where we explain how regional education policies in the Yangtze River Delta (YRD) directly impact the number of joint patent applications, providing clear evidence of policy effectiveness in fostering innovation.  **2. Regional Economic Variability:** To better highlight the regional differences in policy effectiveness, We have incorporated examples from cities like Jiangsu, and Hangzhou. This illustrates how regions with varying economic levels and innovation infrastructures experience different outcomes from the regional education integration policies. We discuss how cities with higher economic levels, such as Shanghai, benefit more from these policies, while less developed regions show more modest results due to resource limitations.  **3. Intensification of Innovation Outputs:** We have expanded on the varying impacts of the policies based on the intensity of innovation outputs. Specifically, we discuss how high-intensity innovation sectors (e.g., high-tech industries) are more significantly influenced by regional education integration policies compared to lower-intensity sectors. This addition helps to contextualize the effectiveness of the policies in different innovation environments.  **4. Key Drivers of UIR Collaboration:** We have provided a deeper analysis of the internal mechanisms that drive UIR collaboration, focusing on the critical roles of human capital, government support, economic capital, and social stability. We further explain how these factors vary across regions in the Yangtze River Delta and their differential impact on innovation outcomes. This helps to connect the theoretical framework of Innovation Systems Theory and Synergetics Theory to the empirical findings.  **5. Global Relevance of Findings:** To address the broader applicability of the study's findings, We have added a discussion on how the insights gained from the Yangtze River Delta region can be relevant to other developing economies. We draw comparisons to other global innovation ecosystems (such as Silicon Valley) to highlight the universal aspects of the policy's impact, suggesting that similar strategies may be effective in other emerging economies.  *Collectively, these revisions significantly enhance the manuscript’s contribution by providing a more nuanced and empirically grounded understanding of how regional education policies influence UIR collaboration, particularly within the context of China’s evolving innovation landscape.*  *Thank you once again for your constructive feedback, which has been instrumental in improving the quality of this work.* |
| ***Results and Analysis***  ***2. Time Series Insights: Present unit root test results to establish the consistency of the data, and interpret the time-fixed effects, explaining how they reflect the evolution of policy impacts over time.*** | *We sincerely appreciate your insightful feedback regarding the time series analysis in our study. Your suggestion to elaborate on the unit root test results and interpret the time-fixed effects has significantly strengthened the robustness and clarity of our empirical analysis.*  To address the first point on unit root tests, we conducted Augmented Dickey-Fuller (ADF) tests on all variables to ensure stationarity. As detailed in Section 5.1.1 (Table 2), the original sequences for variables such as patent, urban, and gap exhibited non-stationarity, but their first-differenced forms rejected the null hypothesis of a unit root at the 1% significance level. This confirms that the variables are integrated of order one, ensuring the validity of our regression models. Additionally, the Engle-Granger cointegration test (Table 3) verified a long-term equilibrium relationship among the variables, further supporting the use of our difference-in-differences (DID) framework.  Regarding the interpretation of time-fixed effects, we expanded the discussion in Section 5.2.1 (Benchmark Model Regression). The inclusion of year fixed effects controls for unobserved temporal shocks—such as macroeconomic cycles or nationwide policy shifts—that could confound the policy impact. For instance, the gradual increase in the DID coefficient post-policy implementation (Figure 2) reflects the dynamic and cumulative nature of the education integration policy’s effects. Specifically, the time-fixed effects capture how policy impacts evolved from initial resource integration (2014–2016) to sustained collaboration growth (2017–2021), aligning with the phased implementation of the Yangtze River Delta’s integration strategy.  *Your suggestion has significantly strengthened our analytical framework by introducing a dynamic dimension to complement the cross-sectional analysis. We believe these enhancements not only address methodological rigor but also provide policymakers with actionable insights about phased investment strategies and impact timelines.* |
| ***Policy Implications***  ***1. Relevant Policy Insights:***  ***o Innovation Ecosystem Support: Recommend policies to create supportive environments for UIR collaborations, such as funding incentives and platforms for industry-academia partnerships.***  ***o Educational Integration Policies: Encourage enhanced integration of educational institutions into innovation-driven economic frameworks, especially in tech hubs and high-growth regions in China.***  ***o Future Directions: Suggest policies that could further regional integration by focusing on digital transformation in educational frameworks, particularly in fields relevant to the emerging needs of the Chinese economy, such as AI and renewable energy.*** | *Thank you for your constructive feedback on the manuscript. We have carefully considered your suggestions, particularly regarding the policy implications section, and have made several revisions to address your concerns in the revised manuscript page 33.*  **1. Innovation Ecosystem Support:** We have added specific recommendations for creating supportive environments for UIR collaborations, including the introduction of multi-level funding incentive schemes, such as tax breaks for enterprises engaging in long-term collaborations with universities, and performance-based funding linked to collaborative outcomes like patents and joint publications.  **2. Educational Integration Policies:** We have emphasized the importance of aligning educational integration policies with sectoral innovation priorities, especially in high-growth regions like the Yangtze River Delta and the Guangdong-Hongkong-Macao Greater Bay Area in China. This ensures that universities are more deeply embedded in regional industrial value chains, which is critical for fostering effective UIR collaborations.  **3. Future Directions and Digital Transformation:** we have included a suggestion for promoting the digital transformation of education systems, particularly by developing interdisciplinary programs related to emerging sectors like artificial intelligence, green technology, and digital manufacturing. This aligns with the evolving needs of China’s innovation economy and offers guidance on future policy directions.  *These additions provide a more comprehensive perspective on the policy implications, offering practical guidance for fostering sustainable innovation-driven growth.*  *Thank you once again for your constructive feedback.* |
| ***Conclusion and Future Scope***  ***1. Summarize Contributions and Limitations: Briefly summarize how the study contributes to understanding the effects of regional educational policies on collaborative innovation, while noting any limitations in scope or methodology.*** | *Thank you very much for your thoughtful feedback and constructive suggestions. We appreciate the opportunity to improve our manuscript and have made several revisions based on your comments. Below is a summary of the modifications we have made, particularly in the conclusion and limitations sections, to better address the issues you raised.*  **1. Contributions and Modifications**  We have revised the Conclusion section to more clearly highlight the key contributions of our research. Specifically, we emphasize how this study enhances the understanding of the impact of regional education integration policies on university-industry-research (UIR) innovation collaboration. We have also added clarity regarding the heterogeneity of policy effects across different regions and innovation output intensities, which is a crucial aspect that has been underexplored in previous literature. Our findings demonstrate that these policies are particularly effective in regions with strong economic foundations and well-developed educational resources, which aligns with the theoretical framework of innovation systems. Furthermore, we highlight the key mediating factors—such as human capital, government expenditure, economic capital, and social stability—that facilitate the positive impact of these policies.  The revised conclusion is as follows in the revised manuscript page 33:  "This study contributes to the understanding of how regional education integration policies influence UIR innovation cooperation. By using panel data from China’s provinces between 2006 and 2021 and applying the DID method, we demonstrate that these policies significantly promote UIR collaboration, especially in regions with strong economic foundations and well-developed educational resources. The study contributes to the literature by highlighting the heterogeneity of policy effects across different provinces and innovation output intensities, which has been underexplored in previous research. The findings suggest that policies are more effective in regions with robust economic fundamentals and where scientific and technological innovation plays a significant role, rather than in regions driven mainly by market forces. Moreover, our mechanism analysis uncovers key mediating factors—human capital, government expenditure, economic capital, and social stability—that facilitate the positive impact of these policies. This study offers valuable insights for policymakers, emphasizing the need to design region-specific policies that can better integrate educational and industrial resources, particularly in economically diverse regions."  **2. Limitations and Modifications**  We have expanded the Limitations section to more explicitly address the scope of the study, including its regional focus, the short- and medium-term time frame, and the potential influence of digital transformation on education. We have specifically discussed the potential risks associated with the geographic focus on the Yangtze River Delta, which may limit the generalizability of the findings to other regions with different socio-economic contexts. We also noted the need for future studies to explore the long-term impacts of regional education integration policies, especially considering the rise of digital education technologies. Finally, we have elaborated on the potential post-treatment divergence risks due to economic shocks, policy spillover, and shifts in China’s macroeconomic strategies, acknowledging the importance of further research to better understand these factors.  The revised limitations section is as follows in the revised manuscript page 34-35:  "This study has three main limitations. First, the data and policy context are primarily focused on specific regions in China, which may limit the generalizability of the findings to other regions with different economic and educational contexts. While the Yangtze River Delta region serves as a representative case, future research should expand the geographic scope to include other regions in China and compare the effects of similar regional education policies in countries with differing industrial structures and policy environments. This would help to validate the broader applicability of the results.  Second, the study focuses mainly on the short- and medium-term effects of regional education integration policies on UIR cooperation, without considering the long-term impact, particularly in light of the digital transformation of education. The rapid development of digital education policies and their interplay with university research, enterprise R&D, and social innovation could have significant long-term effects that are currently underexplored. Future studies should explore how digital education policies and technological advancements influence the sustainability and evolution of UIR collaborations in the long run.  Third, while our difference-in-differences (DID) framework rigorously tests pre-treatment parallel trends, post-treatment divergence risks persist due to three contextual factors: (1) spatially correlated economic shocks (e.g., trade wars, RMB depreciation crises) disproportionately affecting the Yangtze River Delta region; (2) policy spillover contamination from non-YRD provinces adopting similar innovation cluster policies, attenuating treatment effect estimates; and (3) evolving heterogeneity in extended post-treatment periods, exacerbated by China’s shifting macro-strategies like the 'dual circulation' policy. Although robustness checks using staggered DID estimators [86] confirm medium-term effect stability, long-term interpretations beyond five years remain cautiously framed. These contextual factors, including regional economic shocks and macro-strategic shifts, warrant further research to better understand their influence on the long-term impacts of regional educational policies. Furthermore, additional investigations could explore how these factors interact with regional disparities in innovation capacity, particularly in lower-income or less-developed areas, to better capture the nuances of policy effectiveness."  *We believe these revisions address your comments and provide additional clarity on the contributions and limitations of our study. We are confident that these improvements make the paper more robust and informative for policymakers and academics alike.*  *Thank you once again for your valuable feedback.* |
| ***Conclusion and Future Scope***  ***2. Future Scope: Propose avenues for further research, such as expanding to other Asian economies or assessing the long-term impact of digital educational policies on UIR collaborations.*** | *Thank you for your constructive suggestion to elaborate on the future research directions of this study. we have substantially revised the “Future Scope” section to better reflect your expectations.*  *We restructured the original paragraph to directly incorporate the two directions you emphasized: (1) expanding the scope to other Asian economies to examine the universality of policy effects, and (2) exploring the long-term impacts of digital education policies on university-industry-research (UIR) collaborations. In addition, we elaborated on relevant methodologies and research angles that could support these investigations, including the use of high-frequency data and spatial econometric models. These revisions aim to provide a clearer, more forward-looking roadmap for future scholarly inquiry.*  *The revised Content is as follows in the revised manuscript page 35-36:*  “Future research should explore several important avenues to deepen our understanding of the effects of regional educational policies on UIR collaborations. **First, expanding the scope of the study to include other Asian economies or even a global context would provide valuable insights into the universality and boundary conditions of the findings.** Comparing the effects of regional education integration policies in countries with diverse economic structures—such as India, South Korea, and Japan—could reveal whether similar mechanisms and impacts hold across different institutional and cultural contexts. Furthermore, incorporating the experiences of developed economies with advanced educational frameworks could highlight additional insights for optimizing regional education integration policies globally.  **Second, investigating the long-term impact of digital education policies is essential, especially considering the rapid technological transformations shaping education worldwide.** As digital teaching methods, online platforms, and AI-driven tools become more integral to education, future research should assess how these innovations affect UIR collaboration, particularly with regard to technology diffusion and industrial upgrading. Exploring how digital education policies interact with enterprise R&D and university research in the long term could offer a more comprehensive view of the sustainability of UIR collaborations in a digital economy.  **Third, employing high-frequency data and heterogeneity-robust estimators**—such as synthetic DID or dynamic spatial econometric models—could further refine the analysis, enabling scholars to better isolate transient shocks from sustained policy effects. These methodologies could address the biases caused by spatially correlated economic shocks or policy spillover contamination, enhancing the robustness of future studies.  **Finally, exploring inter-regional policy spillovers and the impact of broader macroeconomic strategies**—like China’s "dual circulation" policy—could reveal new insights into the diffusion of innovation across regions and the factors that influence policy effectiveness over time. These studies could also explore how varying innovation capacities across regions, especially in lower-income or less-developed areas, impact the effectiveness of educational integration policies.”  *We hope this enriched content better reflects the global relevance and evolving technological context of UIR research.*  *Thank you again for your valuable feedback.* |
