# Supplementary material for: Assessing the role of regional educational integration policies in fostering university-industry-research innovation: Evidence from China
Source: PLoS One. 2025 Jul 18;20(7):e0325717. doi: 10.1371/journal.pone.0325717 (PMC12273934; doi:10.1371/journal.pone.0325717)
Supplement: Attachment — Submitted filename: Reviewer Comment.pdf [file pone.0325717.s007.pdf]

Review Comment

1. The new version elaborated how the RDD method was applied to exclude the possibility of the policy having spillover effect on other provinces in the control group. But there are two caveats. 1. It only compared 2013 and 2014 of the treatment and control group. Seeing a significant coefficient between 2013 and 2014 for the treatment group while seeing an insignificant coefficient for the control group can at best prove that there is no spillover effect in the early period. It cannot rule out the possibility that there are spillover effects after 2014. The authors also ran RDD regressions on year 2012 and 2013, 2014 and 2015. They claimed the coefficients in the two cases are not significant. But to prove their point, they should extend the comparison to more pairs of years and should do the same for the control group.
2. Section 5, the approach of placebo test is not clearly explained. What regression has the authors run? How did they get the t values of each experimental group? As the authors said, the test can tell if there are any omitted variables, it is crucial for the authors to elaborate how the t-values are generated. Because this approach is unclear, there is no way to know if the authors successfully rule out the possibility that the increase in Y is (partially) driven by other policies in the 4 provinces.
3. Suppose my doubt in comment 1 and 2 can be successfully addressed, there is no need to have Section 5.1.3 Instrumental Variables Approach, 5.1.7 Alternative IV Specifications, and 5.1.9 Control Function Approach since all these approaches relies on strong assumptions of the exogeneity of the IV in the first stage, which cannot be easily removed with higher order lagged terms.
4. The mechanism analysis in Section 6 does not make sense without support of research proving the existence of the causation between mediator and dependent variables. The step, as the authors described, was to find relations between Y and Z (the explanatory variable), and M (the mediator) and Z. But Z causing Y and Z causing M does not necessarily mean Z causing Y through M. To convince readers, the author should cite research proving M causing Y.
